# A general aerosol-assisted biosynthesis of functional bulk nanocomposites

**DOI:** 10.1093/nsr/nwy144

**Published:** 2018-11-23

**Authors:** Qing-Fang Guan, Zi-Meng Han, Tong-Tong Luo, Huai-Bin Yang, Hai-Wei Liang, Si-Ming Chen, Guang-Sheng Wang, Shu-Hong Yu

**Affiliations:** 1 Division of Nanomaterials & Chemistry, Hefei National Laboratory for Physical Sciences at the Microscale, Department of Chemistry, University of Science and Technology of China, Hefei 230026, China; 2 CAS Center for Excellence in Nanoscience, Collaborative Innovation Center of Suzhou Nano Science and Technology, Hefei Science Center, Department of Chemistry, University of Science and Technology of China, Hefei 230026, China; 3 School of Chemistry and Environment, Beihang University, Beijing 100191, China

**Keywords:** bacterial cellulose, nanoscale building blocks, biosynthesis, electromagnetic shielding, scalable fabrication

## Abstract

Although a variety of nanoparticles with better-than-bulk material performances can be synthesized, it remains a challenge to scale the extraordinary properties of individual nanoscale units to the macroscopic level for bulk nanostructured materials. Here, we report a general and scalable biosynthesis strategy that involves simultaneous growth of cellulose nanofibrils through microbial fermentation and co-deposition of various kinds of nanoscale building blocks (NBBs) through aerosol feeding on solid culture substrates. We employ this biosynthesis strategy to assemble a wide range of NBBs into cellulose nanofibril-based bulk nanocomposites. In particular, the biosynthesized carbon nanotubes/bacterial cellulose nanocomposites that consist of integrated 3D cellulose nanofibril networks simultaneously achieve an extremely high mechanical strength and electrical conductivity, and thus exhibit outstanding performance as high-strength lightweight electromagnetic interference shielding materials. The biosynthesis approach represents a general and efficient strategy for large-scale production of functional bulk nanocomposites with enhanced performances for practical applications. Industrial-scale production of these bulk nanocomposite materials for practical applications can be expected in the near future.

## INTRODUCTION

Over the past decades, significant advances in nanotechnology have provided a variety of nanostructured materials with unique properties, including 0D quantum dots [1], 1D nanowires and nanotubes [2], and 2D nanosheets [3]. Although these nanomaterials, with superior performance to bulk materials in their electronic, magnetic, thermal, and mechanical properties, have been exploited in nearly every field of materials, it is still highly challenging to scale their extraordinary nanoscale properties to the macroscopic level, which has long been recognized as the core issue for practical applications [4,5]. Assembling nanoscale building blocks (NBBs) into bulk materials (e.g. films, aerogels, etc.) that maintain the unique characteristics of individual units would supply enhanced and scalable performance [4,6–8]. An additional benefit of assembly is the emergence of ‘collective' properties and unexpected functionalities that are not ascribable to the individual units [5,7,9,10]. Furthermore, the assemblies frequently go to hybrid structures with multi-components, e.g. incorporation of nanoparticles into a polymer matrix, which enables the creation of materials with new or improved properties by exploiting synergistic effects [6,8,11].

The main assembly strategy for generating bulk nanocomposites is based on the post-synthesis processing of nanoparticle suspensions or nanoparticle–polymer blends [12]. For example, chemical or physical crosslinking in solution offers a direct method to convert nanoscale units into self-supporting hydrogels or aerogels [13,14]. Membrane or paper-like materials of polymer nanocomposites with enhanced mechanical and conductive properties could be prepared by casting or spin-coating of carefully processed nanoparticle–polymer blends [11,15,16]. Although these solution-processing strategies are certainly promising, the challenge lies in the ambiguity of the key factors that influence the particle–polymer interactions and the suspension stability [16–18].

**Figure 1.**
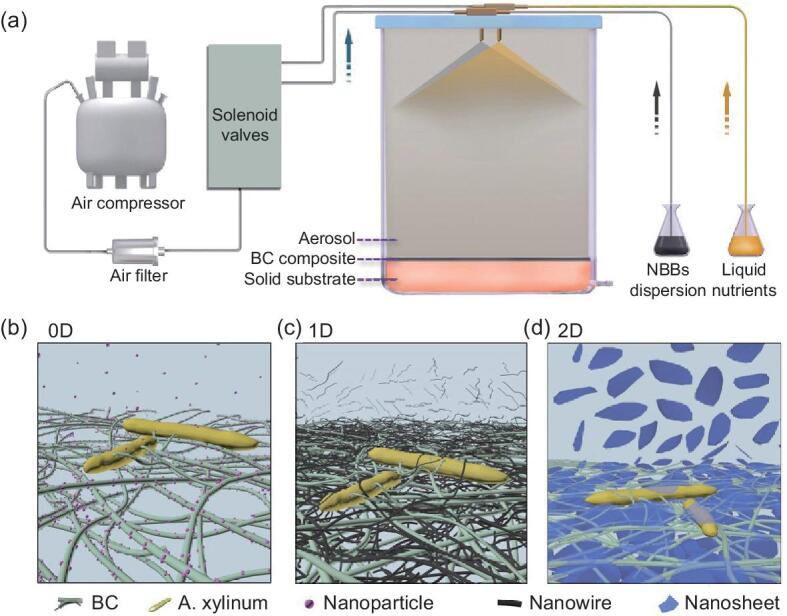
Schematic illustration of the aerosol-assisted biosynthesis of functional bulk nanocomposites. (a) Scheme of the bioreactor. Aerosols of liquid nutrients and nanoscale building block suspension were fed into the bioreactor with filtered compressed air, which was controlled by an automatic control system. (b)–(d) Schematic illustration of the formation uniform BC-based nanocomposites with 0D nanoparticles (b), 1D nanotubes or nanowires (c), and 2D nanosheets (d).

**Figure 2.**
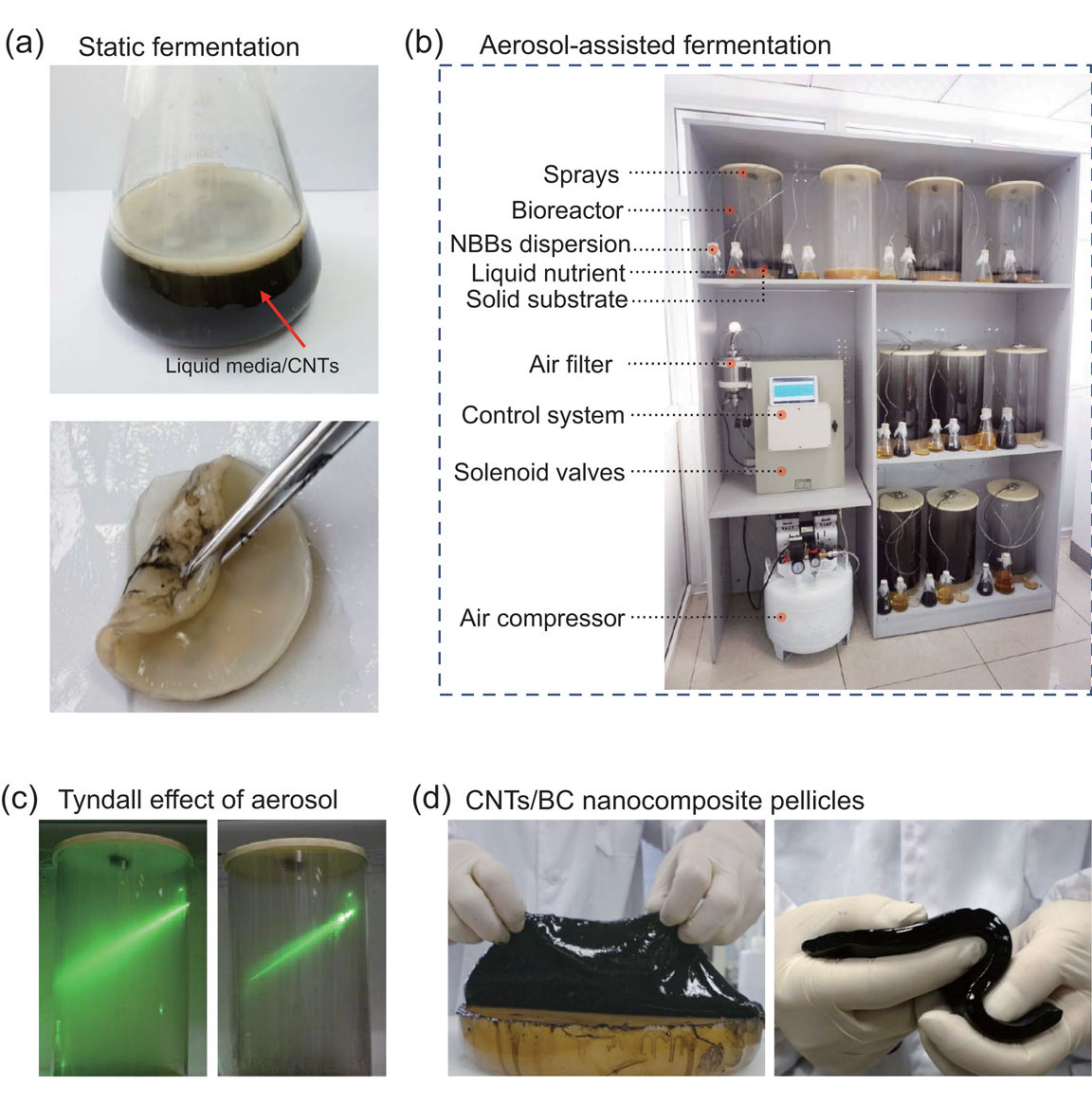
Comparison between traditional static fermentation and aerosol-assisted biosynthesis. (a) Static fermentation in the liquid nutrient dispersed with CNTs. The static fermentation in the liquid media failed to produce CNT/BC nanocomposite. (b) Experimental apparatus. Air is compressed by the air compressor and flows through the air filter to remove microorganisms. The liquid nutrients or NBB suspension are siphoned to the sprays and atomized into aerosol droplets with the compressed and filtered air, which is controlled by the solenoid valves and operated by the control system. (c) Tyndall effect of the aerosol. The Tyndall effect was observed after the spray of liquid nutrients and NBB suspension. (d) Photographs of CNT/BC nanocomposite pellicles. Uniform CNT/BC nanocomposite pellicle could be harvested directly from the solid substrate and the biosynthesized CNT/BC nanocomposites are flexible and mechanically robust.

As a crystalline cellulosic polymer, bacterial cellulose (BC) nanofibrils possess a high tensile strength (almost the same as steel and Kevlar) and spontaneously form a robust 3D nanofibrous network [19], which makes them an ideal platform for the design of functional bulk nanocomposites [8,20,21]. The conventional process of fabricating uniform BC nanocomposites involves the disintegration of 3D network structure for solution processing, which, however, seriously impairs the mechanical performance of the nanocomposites [22]. Pure BC can be manufactured nowadays in large quantities at low cost via a fermentation process in the food industry [23]. Nevertheless, the method for industrial-scale production of BC based on static fermentation failed to produce BC nanocomposites due to the diffusion limitation of nanoscale units from the liquid medium to the upper surface layer of newly grown BC. So far only millimeter-sized, spherical or flocculent BC nanocomposites rather than bulk materials could be prepared on a small scale by agitated fermentation in laboratories [24–27]. We overcome these limitations and achieve scalable mass production of bulk BC-based nanocomposites, which consist of integrated 3D cellulose nanofibril networks, by a biosynthesis system composed of a solid culture substrate and a continuous aerosol feeding system (Figs 1a and 2). The aerosol bioreactor can automatically generate an aerosol spray of NBB suspension and nutrient solution that are both fed directly to the living bacteria on the medium–air interface. The solid culture substrate can effectively avoid the disturbance of the medium–air interface during the fermentation process and guarantees the formation of a uniform NBB/BC nanocomposite pellicles. As expected, our efforts at static fermentation in a conical flask failed to produce NBB/BC nanocomposites (Fig. 2a). Note that the aerosol feeding has been reported previously for the production of pure BC [28].

## RESULTS AND DISCUSSION

### Biosynthesis of functional bulk nanocomposites and larger-scale fabrication

The first step of the aerosol-assisted biosynthesis was growth of a thin film of pure BC onto a solid agar substrate with a bacterial strain, i.e. *Gluconacetobacter xylinus* 1.1812, for 24 h in a cylindrical bioreactor (Fig. 1a). Then, continuous and stable aerosol was produced by an intermittent spray of liquid nutrient and nanoscale building block suspension with filtered compressed air; these were fed on to the top of the bioreactor (Fig. 2b, c). The spray interval was optimized as 30 min according to the electrical conductivity of the BC/carbon nanotube (CNT) composite (Supplementary Fig. 1). The whole spray process was controlled by an automatic control system. With the settling of the aerosol, the NBBs reaching the medium–air interface were entangled by the cellulose microfibrils that were secreted by the bacteria to form a uniform nanocomposite (Fig. 1b–d). After continuous fermentation for 5 to 7 d, wet pellicles with a thickness of ca. 10 mm that consisted of NBBs and cellulose nanofibrils were harvested from the solid substrate (Fig. 2d).

**Figure 3.**
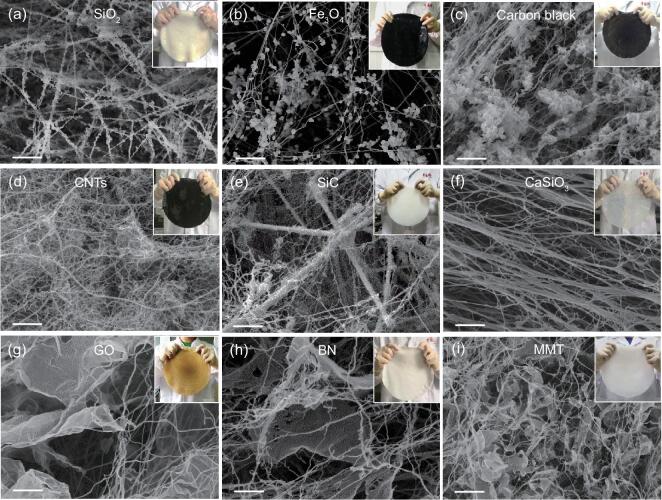
SEM and photographs of BC-based nanocomposites. (a) Silica colloids/BC. (b) Fe_3_O_4_ nanoparticles/BC. (c) Carbon black/BC. (d) CNTs/BC. (e) SiC nanowires/BC. (f) CaSiO_3_ nanowires/BC. (g) GO nanosheets/BC. (h) BN nanosheets/BC. (i) MMT nanosheets/BC. All scale bars are 1 μm.

To demonstrate the versatility of the aerosol-assisted biosynthesis, we assembled a variety of NBBs into cellulose nanofibril-based nanocomposites by the biosynthesis strategy (Fig. 3), including 0D carbon black, SiO_2_ and Fe_3_O_4_ microspheres; 1D carbon nanotubes (CNTs), CaSiO_3_ and SiC nanowires; and 2D graphene oxide (GO), BN and montmorillonite (MMT) nanosheets (Supplementary Fig. 2). Macroscopically, the synthesized nanocomposites are mechanically robust wet gels with different colors depending on the NBBs (Fig. 3, insets). Scanning electron microscopy (SEM) observations of the freeze-dried gels revealed that the NBBs were uniformly mixed with cellulose nanofibrils (Fig. 3 and Supplementary Fig. 3). Specifically, the 0D nanoparticles tended to be absorbed onto the surface of the cellulose nanofibrils (Fig. 3a–c and Supplementary Fig. 3a–c), while the 1D nanowires/nanotubes were intertwined with the cellulose nanofibrils (Fig. 3d–f and Supplementary Fig. 3d–f) and the 2D nanosheets were randomly distributed throughout the whole nanofibril network (Fig. 3g–i and Supplementary Fig. 3g–i).

When the microfibrils (3–7 nm in diameter) of a glucose chain are secreted through a bacteria cell wall, they tend to aggregate together through strong hydrogen bonds to form wide cellulose nanoribbons to reduce their high surface energy [19]. The incorporation of NBBs disrupted the bundling of microfibrils into ribbons during biosynthesis and resulted in thinner cellulose fibrils in the composites compared to a pure BC sample. For example, the addition of MMT interrupted the crystallization of BC, leading to a higher transparency of the MMT/BC hydrogel and film compared to pure BC samples as a result of the thinner BC nanofibrils in MMT/BC (Supplementary Figs 4 and 5). A similar phenomenon of size reduction of BC was also observed for the case of SiO_2_/BC (Supplementary Fig. 6). X-ray powder diffraction (XRD) analyses showed decreased ratios of the (101)/(002) peaks of the cellulose in the composites, further confirming the disruption of NBBs on the bundling of microfibrils in the biosynthesis (Supplementary Figs 7 and 8).

**Figure 4.**
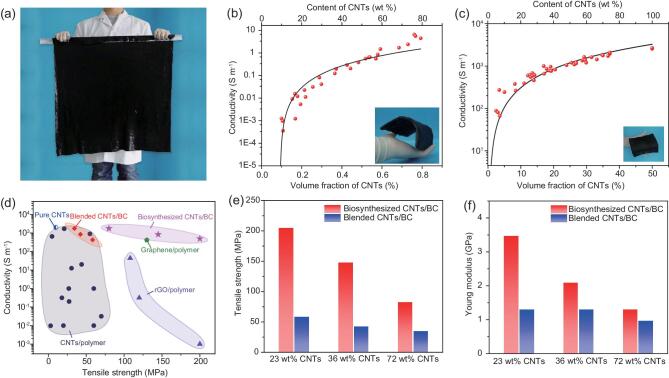
Large-scale biosynthesis of CNT/BC bulk nanocomposites and their properties. (a) Photograph of a large-sized CNT/BC pellicle with a volume of 800 × 800 × 8 mm^3^. (b) and (c) Electrical conductivity of the CNT/BC aerogels (b) and films (c) as a function of CNT volume and weight fraction. The black lines are predictions based on a power-law relationship (Eq. (1) in the main text) and 3D percolation theory. The insets are photographs of CNT/BC aerogel and film, respectively. (d) Comparison of the electrical conductivity and tensile strength of the biosynthesized CNT/BC nanocomposites with blended CNT/BC nanocomposites and reported CNT, reduced graphene oxide (RGO), and graphene-based polymer nanocomposites. Each symbol indicates a material category set. A detailed description of each data point is presented in Supplementary Table 1. (e) and (f) Comparison of the tensile strength (e) and Young modulus (f) between the biosynthesized and blended CNT/BC nanocomposites, demonstrating the advantages of the biosynthesis strategy for preparing mechanically reinforced nanocomposite.

The aerosol-assisted biosynthesis could be easily scaled up for potentially industrial applications by using large reactors and increasing the number of nozzles. For example, we fabricated a large-sized CNT/BC composite gel with a volume of 800 × 800 × 8 mm^3^ (Fig. 4a) by using a large reactor equipped with 18 nozzles (Supplementary Fig. 9a). Thermogravimetric analysis (TGA) analyses verified that the distribution of CNTs throughout the whole nanocomposite gel was highly uniform (Supplementary Fig. 9b, c).

### Properties of biosynthesized functional bulk nanocomposites

Benefiting from the uniform distribution of NBBs in the biosynthesized nanocomposites, the extraordinary properties of the NBBs as well as the cellulose nanofibrils were successfully scaled up to the macroscopic level. For example, freeze-drying of the CNT/BC composite gel resulted in an electrically conductive, ultralight, and mechanically robust aerogel (Fig. 4b, inset). Importantly, the CNT content in the composites could be easily tuned in a wide range from 1.5 wt% to 75 wt% by changing the concentration of the CNT suspension. The conductivity of a nanocomposite with rigid filler particles (e.g. CNTs) is typically described with a power-law relationship and 3D percolation theory as [29]:

(1) \begin{equation*} \sigma = {\sigma _0}{\left( {v - {v_c}} \right)^t}, \end{equation*}

where σ is the conductivity of the composite, σ_0_ is a scaling factor proportional to the intrinsic conductivity of the filler, *υ* is the volume fraction of conductive filler, *υ_c_* is the volumetric fraction at the percolation threshold, and *t* is the critical exponent of the conductivity. The best fitting of the conductivity data to the laws of power give a percolation threshold of ∼0.1 vol.% and a critical exponent of ∼2.1 according to Eq. (1) in the CNT/BC aerogels (Fig. 4b). Remarkably, the conductivity of the CNT/BC composite aerogels reached ca. 1.5 and 6.0 S m^−1^ for the samples with 60 wt% and 75 wt% CNTs, respectively, which is higher than that of PVA-reinforced CNT aerogels (10^−3^ S m^−1^) [13] and comparable to that of CNT-modified nanofibrillated cellulose aerogels (1.0 S m^−1^) [30]. Note that monolithic CNT sponges with high conductivity (ca. 160 S m^−1^) could be prepared by the chemical vapor deposition (CVD) method [31].

Besides the aerogels, mechanically reinforced and highly conductive CNT/BC films (Fig. 4c, inset) were easily produced by directly hot-pressing the nanocomposite pellicles under a pressure of 100 MPa at 100°C for 20 min. The conductivity of the CNT/BC film increased rapidly with the CNT contents until reaching ca. 2100 S m^−1^ for the nanocomposite film with 75 wt% CNTs (Fig. 4c). The estimated percolation threshold according to the power-law relationship is ∼0.5 vol% for the CNT/BC nanocomposite films, which is consistent with the values of reported CNT/polymer composites [32]. Interestingly, the CNT/BC films exhibited a lower critical exponent (*t* = 1.4) than that of CNT/BC aerogels (*t* = 2.1), which can be explained by the reduction of ‘dead arms' of the CNT networks present in the CNT/BC gels when hot-pressing them into dense film samples [33].

The mechanical properties of the CNT/BC films were also investigated as a function of CNT content. The stress–strain curves of the pure BC and CNT/BC composite films clearly indicated that both of them showed typical brittle properties and no yielding point was observed (Supplementary Fig. 10). Although the Young's modulus and the tensile strength of the CNT/BC composite films decreased with increasing CNT content due to the reduced hydrogen-bonding interaction after the incorporation of CNTs (Fig. 4e, f and Supplementary Fig. 10), an ideal balance of the mechanical strength and conductivity of the composite films could be achieved for practical applications by controlling the CNT content in the composites (Fig. 4d). Remarkably, the composite film with 23 wt% CNTs showed a high tensile strength of 207 MPa as well as a high electrical conductivity of ca. 500 S m^−1^; the conductivity of the composite film with 36 wt% CNTs increased to ca. 830 S m^−1^; meanwhile the tensile strength was still higher than 148 MPa. These values are much higher than the reported values for CNTs and graphene nanocomposites (Fig. 4d and Supplementary Table 1). The conventional fabrication method for CNT nanocomposites that requires the mixing of CNT dispersions with polymer solutions is only applicable to the preparation of polymer nanocomposites with low CNTs (< 10 wt%), as it is extremely difficult to homogeneously disperse high-concentration CNTs in polymeric hosts [32]. In our work, such limitations were overcome by feeding a large quantity of CNT aerosol into a bioreactor that produced crystalline cellulosic polymer nanofibers; the CNT content in the nanocomposites could be up to 75 wt%, resulting in a highly conductive nanocomposite with considerable mechanical strength.

To demonstrate the advantages of the present biosynthesis strategy for preparing mechanically reinforced nanocomposites, CNT/BC nanocomposite films were also prepared for comparison by blending CNTs and disintegrated BC suspensions with a mixer into a homogeneous mixture, followed by filtration and hot-pressing of the mixture under the same conditions (Supplementary Fig. 11). The conductivities of the blended CNT/BC films were almost the same as that of biosynthesized CNT/BC films over the full range of CNT content, further confirming the highly uniform distribution of CNTs in the biosynthesized samples (Supplementary Fig. 12). More importantly, both the tensile strength and Young's modulus of the biosynthesized CNT/BC nanocomposites were remarkably higher than the blended samples (Fig. 4d–f). In particular, the tensile strength and Young's modulus of the biosynthesized CNT/BC film with 23 wt% CNTs were 3.5 and 2.7 times that of blended CNT/BC film with the same CNT content, respectively. It is believed that the integrated long BC nanofibril network in the biosynthesized nanocomposites contributes their enhanced mechanical properties [34], which was further verified by the fact that the directly hot-pressed pure BC films exhibited much higher tensile strength than the film sample fabricated from pure disintegrated BC suspension (Supplementary Fig. 13).

**Figure 5.**
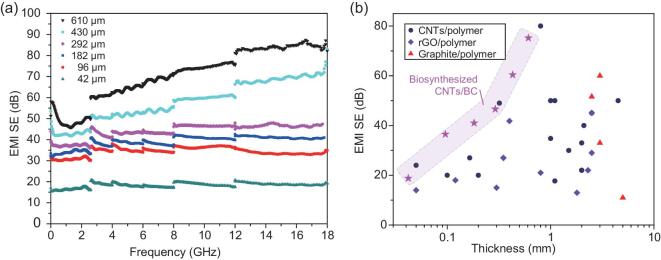
EMI SE of CNT/BC bulk nanocomposites. (a) EMI SE of the biosynthesized CNT/BC nanocomposite films at different thicknesses. (b) Comparison of EMI SE with reported CNT, RGO, and graphite-based polymer nanocomposites. Each symbol indicates a material category set. A detailed description of each data point is presented in Supplementary Table 2.

Based on the outstanding electrical conductivity and mechanical properties, the electromagnetic interference (EMI) shielding properties of the CNT/BC nanocomposite films with a high electrical conductivity of ∼1300 S m^−1^ (57 wt% CNTs) were investigated over a broad frequency range from 3 × 10^−4^ to 18 GHz (Fig. 5a). Clearly, our biosynthesized CNT/BC nanocomposite films exhibited attractive shielding performance over all the measured frequency region, and the EMI shielding effectiveness (SE) increased from about 18 to 75 dB in the X-band as the thickness increased from 42 to 610 μm. Although adequate shielding can be achieved by using thick conventional materials, lightweight and mechanically robust film samples are advantageous for use in aerospace and telecommunication applications [35]. Taking the density and thickness into account, the CNT/BC nanocomposite films were significantly superior to most carbon/polymer composites for EMI shielding reported to date (Fig. 5b and Supplementary Tables 2 and 3). Note that even though the CNT/polyurethane nanocomposites exhibited a high EMI SE of 80 dB at a thickness of 800 μm [36], its tensile strength (only 2.6 MPa) is not satisfied for many practical applications and is much lower than that of our CNT/BC nanocomposite films (108 MPa).

Despite the fact that we are currently focusing on CNT-based nanocomposite aerogels and films in this work, all the biosynthesized pellicles can be converted into corresponding functional bulk nanocomposites. For example, the biosynthesized Fe_3_O_4_/BC nanocomposite films exhibited superparamagnetic behavior and high tensile strength and are expected to be useful as flexible electromagnetic actuators (Supplementary Fig. 14).

## CONCLUSION

In this study, we have reported a general and scalable biosynthesis strategy that involves simultaneous growth of cellulose nanofibrils through microbial fermentation and co-deposition of various kinds of NBBs through aerosol feeding on solid culture substrates. A wide range of cellulose nanofibril-based bulk nanocomposites with high performance was prepared by the biosynthesis strategy. The biosynthesized carbon nanotube/bacterial cellulose nanocomposites that consist of integrated 3D cellulose nanofibril networks simultaneously achieve an extremely high mechanical strength and electrical conductivity, and thus exhibit outstanding performance as high-strength lightweight electromagnetic interference shielding materials. By upgrading the state-of-the-art production line that produces pure BC pellicles, industrial-scale production of these bulk nanocomposite materials for practical applications can be expected in the near future.

## METHODS

### Solid substrate and liquid nutrient media preparation

All reagents were a commercially available. 1.0 L aqueous mixture consisting of glucose (50 g L^−1^), yeast extract (5 g L^−1^), CaCO_3_ (10 g L^−1^), and agar (20 g L^−1^); this was heated and stirred to fully dissolve all components, and then sterilized in an autoclave at 121°C for 30 min. The mixture was cooled naturally until a solid plate with a height of about 1.0 cm was formed. The diameter and height of the typical cylindrical bioreactors is 24 and 50 cm, respectively (Supplementary Figs 1 and 10).

1.0 L liquid nutrient media consisted of 50 g L^−1^ glucose, 5 g L^−1^ yeast extract, 2 g L^−1^ citric acid, 4 g L^−1^ Na_2_HPO_4_·12H_2_O, and 2 g L^−1^ KH_2_PO_4_. The media were sterilized in an autoclave at 121°C for 30 min before inoculation and biosynthesis.

### Nanoscale building block suspension preparation

Silica particles (LUDOX® SM-30) were purchased from Aldrich. The suspension was diluted into 30.0 mg mL^-1^ and sterilized in an autoclave at 121°C for 30 min.

The Fe_3_O_4_ nanoparticles were prepared according to the reported protocol [37]. In a typical process, 1.35 g FeCl_3_·6H_2_O was dissolved in 40 mL ethylene glycol followed by the addition of 3.6 g NaAc and 1.0 g glycol. Then the mixture was stirred for 30 min and transferred into a Teflon-lined stainless-steel autoclave (50 mL in total volume), which was heated at 200°C for 8 h before cooling down naturally. The product was washed and dispersed into sterile water to form a homogeneous suspension (15 mg mL^−1^).

The carbon black suspension was prepared by dispersing acetylene carbon black (STREM Chemicals Inc., USA) into sterile water with a concentration of 2.0 mg mL^−1^.

The CNT dispersion (TNWDM-MC2, 2.0 wt% CNTs, 1.2 wt% dispersing agent) was purchased from Chengdu Organic Chemicals Co., Ltd (China). The dispersion was diluted into 0.5, 1.0, 2.0, 4.0, 6.0, 8.0, 10.0, or 12.0 mg mL^−1^ for preparing CNT/BC nanocomposites with various CNT contents.

The SiC nanowire suspension was prepared by homogeneously dispersing CVD SiC nanowire (Changsha Sinet Advanced Materials Co., Ltd, China) into sterile water with a concentration of 10.0 mg mL^−1^.

The CaSiO_3_ nanowire was prepared according to the reported method [38]. In a typical process, 40 mL Ca(NO_3_)_2_ solution (0.5 M) was added dropwise into 40 mL Na_2_SiO_3_ solution (0.5 M) at room temperature under stirring to form a homogeneous mixture. The mixed solution was then transferred into a Teflon-lined stainless-steel autoclave (100 mL in total volume) and heated at 200°C for 24 h before cooling down naturally. The product was washed by distilled water and anhydrous ethanol several times before vacuum filtration and dried at 120°C for 24 h. The dried product was dispersed into sterile water to form a CaSiO_3_ nanowire suspension with a concentration of 5.0 mg mL^−1^.

The GO was prepared via a modified Hummers method [39,40]. The concentration of the GO suspension was 1.0 mg mL^−1^.

The BN nanosheet suspension was prepared by homogeneously dispersing BN nanosheet (Nanjing XFNANO Materials Tech Co., Ltd, China) into sterile water with a concentration of 1.0 mg mL^−1^.

Sodium montmorillonite (MMT) clays were kindly offered by the Zhejiang Fenghong Clay Co., Ltd, China. For the exfoliation of MMT into nanosheets, 10 g MMT was dispersed in 2.0 L deionized water under vigorous stirring for 1 wk. Afterwards, the supernatant was collected as an MMT nanosheet solution for further use. The concentration of the MMT nanosheet suspension for biosynthesis was 2.0 mg mL^−1^.

Glass bubbles (GBs) were purchased from the 3M company. They were dispersed into sterile water to form a homogeneous suspension with a total GB content of 3.0 mg mL^−1^.

### Inoculation

The pre-inocula for all experiments were thin films prepared by inoculating *Gluconacetobacter xylinus* 1.1812 (China General Microbiological Culture Collection Center, CGMCC) onto an agar culture media plate at 30°C for 48 h. The thin films were transferred onto the solid substrates in the bioreactors. A brush was used to ensure uniform and complete coverage of the pre-inoculum over the whole substrate.

### Aerosol-assisted biosynthesis

The agar culture media solid substrate was first inoculated for 24 h to form a pure BC thin film. Afterwards, continuous aerosols of liquid nutrients and nanoscale building block suspension were produced by intermittent sprays of liquid nutrients and nanoscale building block suspension with filtered compressed air and fed into the top of the bioreactor. The whole spray process was controlled by an automatic control system (Fig. 1). The spray duration was 4 s and the interval was 30 min for a typical process. After continuous fermentation for 5 to 7 d, nanocomposite pellicles with a thickness of ca. 10 mm, which were composed of uniformly distributed nanoscale building blocks and cellulose nanofibrils, were harvested from the solid substrate. The harvested pellicles were then soaked with 2 wt% NaOH solution and boiled for 30 min, and finally rinsed with water to neutralize them.

### Nanocomposite aerogel and film preparation

Nanoscale building block/BC aerogels were produced by freeze-drying or CO_2_ supercritical drying of the harvested purified pellicles. Nanocomposite films were produced by direct hot-pressing of the purified wet pellicles under a pressure of 100 MPa at 80°C for 10 min.

### Blended CNT/BC nanocomposite film fabrication

Pure BC pellicles were cut into pieces and disintegrated by a blender. The BC suspension and CNT dispersion in different ratios were mixed by vigorous stirring for 30 min, and then vacuum-filtered to obtain wet film. The wet films were hot-pressed under a pressure of 100 MPa at 80°C for 10 min.

### Sample characterizations

SEM images were taken with a Carl Zeiss Supra 40 field emission scanning electron microscope (2–5 kV, depending on the sample state). All samples were measured in the form of aerogels that were prepared by freeze-drying of the purified wet nanocomposite pellicles. All aerogel samples were sputtered with gold for 30 s at a constant current of 30 mA before observation. Transmission electron microscopy (TEM) images were acquired using a JEOL JEM-ARM200F transmission electron microscope (200 kV). XRD data were measured by a PANalytical X'pert PRO MRD X-ray diffractometer equipped with Cu Kα radiation (λ = 1.54056 Å). The samples were tested in the form of films prepared by hot-pressing of the purified wet nanocomposite pellicles. UV–vis spectra were recorded on UV-2501PC/2550 at room temperature (Shimadzu Corporation, Japan). TGA data were measured in a nitrogen atmosphere with a TA Instruments SDT Q600 thermogravimetric analyzer. All the samples were tested in the form of films.

### Tensile tests

Tensile tests were performed on an Instron 5565A universal testing machine. The samples were tested in the form of film strips with a size of 2 mm × 25 mm. The test was carried out at room temperature with a displacement rate of 1.0 mm min^−1^.

### Electrical conductivity measurements

The electrical conductivity of the CNT/BC aerogels and films was measured by a two-probe method with a multimeter at room temperature in air. A layer of Ag paste was uniformly pasted on two opposite sides of the samples as electrode pairs.

### Electromagnetic shielding effectiveness

The measurement at the frequency of 300 KHz–2.6 GHz was performed by the flange coaxial method and the diameter of the sample was 133 mm. The measurement at the frequency of 2.6–18 GHz was performed by TE 10 waveguide techniques. The sample sizes for the measurement by the TE 10 waveguide techniques are shown in Table 1.

**Table 1.** The sample sizes for the measurement at different frequency.

Frequency (GHz)	Sample size (mm^2^)
2.6–3.95	114 × 76
3.95–5.85	89 × 64
5.85–8.2	68.5 × 49
8.2–12.4	41.5 × 41.5
12.4–18	33.5 × 33.5

### Magnetic property measurements

The magnetic properties of the samples were investigated using a superconducting quantum interface device (SQUID) magnetometer (Quantum Design MPMS XL).

### Calculation of specific shielding effectiveness (SSE)

The SSE was obtained by dividing the EMI SE by the density of the material [41,42]:

(2) \begin{equation*} {\rm{SSE}} = {\rm{EMI}}\,\,{\rm{SE}}/{\rm{density}} = {\rm{dB}}\,\,{\rm{c}}{{\rm{m}}^3}\,\,{{\rm{g}}^{ - 1}} \end{equation*}

Accounting for the contribution of thickness (*t*), the absolute effectiveness (SSE*_t_*) was used [41,42]:

(3) \begin{equation*} {\rm{SS}}{{\rm{E}}_t} \!=\! {\rm{SSE}}/t = {\rm{dB}}\,\,{\rm{c}}{{\rm{m}}^3}\,\,{{\rm{g}}^{ - 1}}\,\,{\rm{c}}{{\rm{m}}^{ - 1}} = {\rm{dB}}\,\,{\rm{c}}{{\rm{m}}^2}\,\,{{\rm{g}}^{ - 1}} \end{equation*}

### Calculation of the CNT content through TGA data

The CNT content in the biosynthesized CNT/BC nanocomposite was estimated based on the TGA data:

(4) \begin{equation*} {\emptyset _0} \cdot \ {m_0} = {\emptyset _{{\rm{CNT}}}} \cdot {m_{{\rm{CNT}}}} + {\emptyset _{{\rm{cellulose}}}} \cdot {m_{{\rm{cellulose}}}}, \end{equation*}

where ${\emptyset _0}$ is the total residual ratio of TGA at nitrogen atmosphere, ${m_0}$ is the total mass of composite, ${\emptyset _{{\rm{CNT}}}}$ is the residual ratio of pure CNT under the same test conditions, ${m_{{\rm{CNT}}}}$ is the CNT mass in the composite, ${\emptyset _{{\rm{cellulose}}}}$ is the residual ratio of pure bacterial cellulose under the same TGA measurement conditions and ${m_{{\rm{cellulose}}}}$ is the mass of cellulose in the composite:

(5) \begin{equation*} \begin{array}{@{}*{1}{l}@{}} {\ {m_0} = \ {m_{{\rm{cellulose}}}} + {m_{{\rm{CNT}}}}}\\ {w{t_{{\rm{CNT}}}}\% = \displaystyle\frac{{{m_{{\rm{CNT}}}}}}{{{m_0}}}\ \times \ 100\% = \displaystyle\frac{{{\emptyset _0} - {\emptyset _{{\rm{cellulose}}}}}}{{{\emptyset _{{\rm{CNT}}}} - {\emptyset _{{\rm{cellulose}}}}}}} \\ \, {\times\, 100\% } \\ \end{array} \end{equation*}

## Supplementary Material

Supplemental File
