# Supplementary material for: A general aerosol-assisted biosynthesis of functional bulk nanocomposites
Source: Natl Sci Rev. 2018 Nov 23;6(1):64–73. doi: 10.1093/nsr/nwy144 (PMC8291477; doi:10.1093/nsr/nwy144)
Supplement: Supplemental File [file nwy144_supplemental_file.docx]

Supplementary Information

A general aerosol-assisted biosynthesis of functional bulk nanocomposites

Qing-Fang Guan^1^, Zi-Meng Han^1^, Tong-Tong Luo^1^, Huai-Bin Yang^1^, Hai-Wei Liang^1^, Si-Ming Chen^1^, Guang-Sheng Wang^3^ & Shu-Hong Yu^1,2^

*^1^ Division of Nanomaterials & Chemistry, Hefei National Laboratory for Physical Sciences at the Microscale, Department of Chemistry, University of Science and Technology of China, Hefei, 230026, China.*

*^2^ CAS Center for Excellence in Nanoscience, Collaborative Innovation Center of Suzhou Nano Science and Technology, Hefei Science Center, Department of Chemistry, University of Science and Technology of China, Hefei, 230026, China.*

*^3^ School of Chemistry and Environment, Beihang University, Beijing 100191, China.*

*Correspondence and requests for materials should be addressed to Shu-Hong Yu ([shyu@ustc.edu.cn](mailto:shyu@ustc.edu.cn)) and Hai-Wei Liang ([hwliang@ustc.edu.cn](mailto:hwliang@ustc.edu.cn)).

**Supplementary Figures**


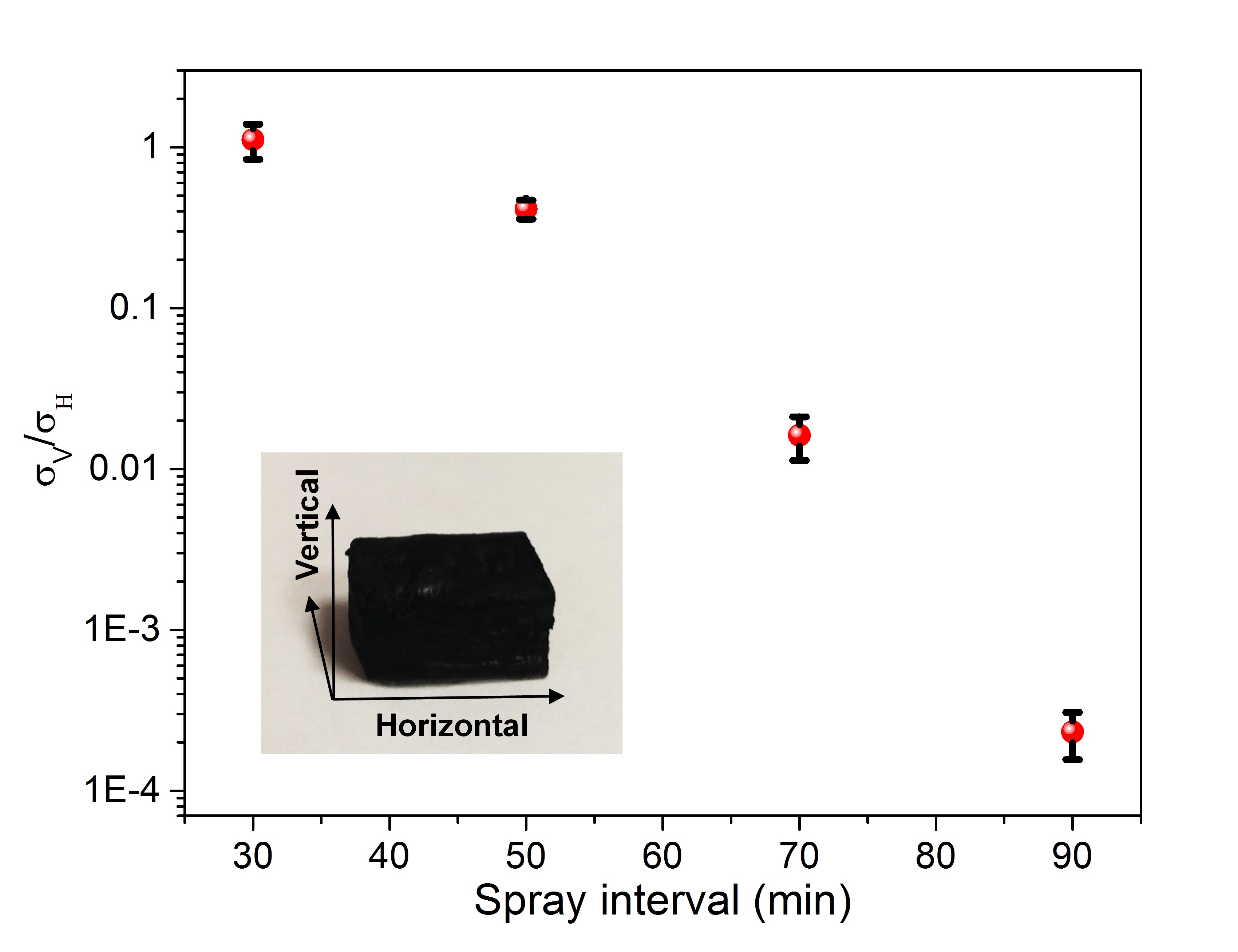


**Supplementary Figure 1. Ratio of the conductivities between the vertical and horizontal direction as a function of the spray interval of CNTs suspension.** The spray interval was optimized to be 30 min for the biosynthesis, at which the conductivity of the CNTs/BC nanocomposite aerogels along the vertical direction is the same with that along the horizontal direction. Longer spray interval resulted in much lower conductivity along the vertical direction, indicating the non-uniform distribution of CNTs in the nanocomposite along the vertical direction due to the decreased CNTs concentration in the aerosol.


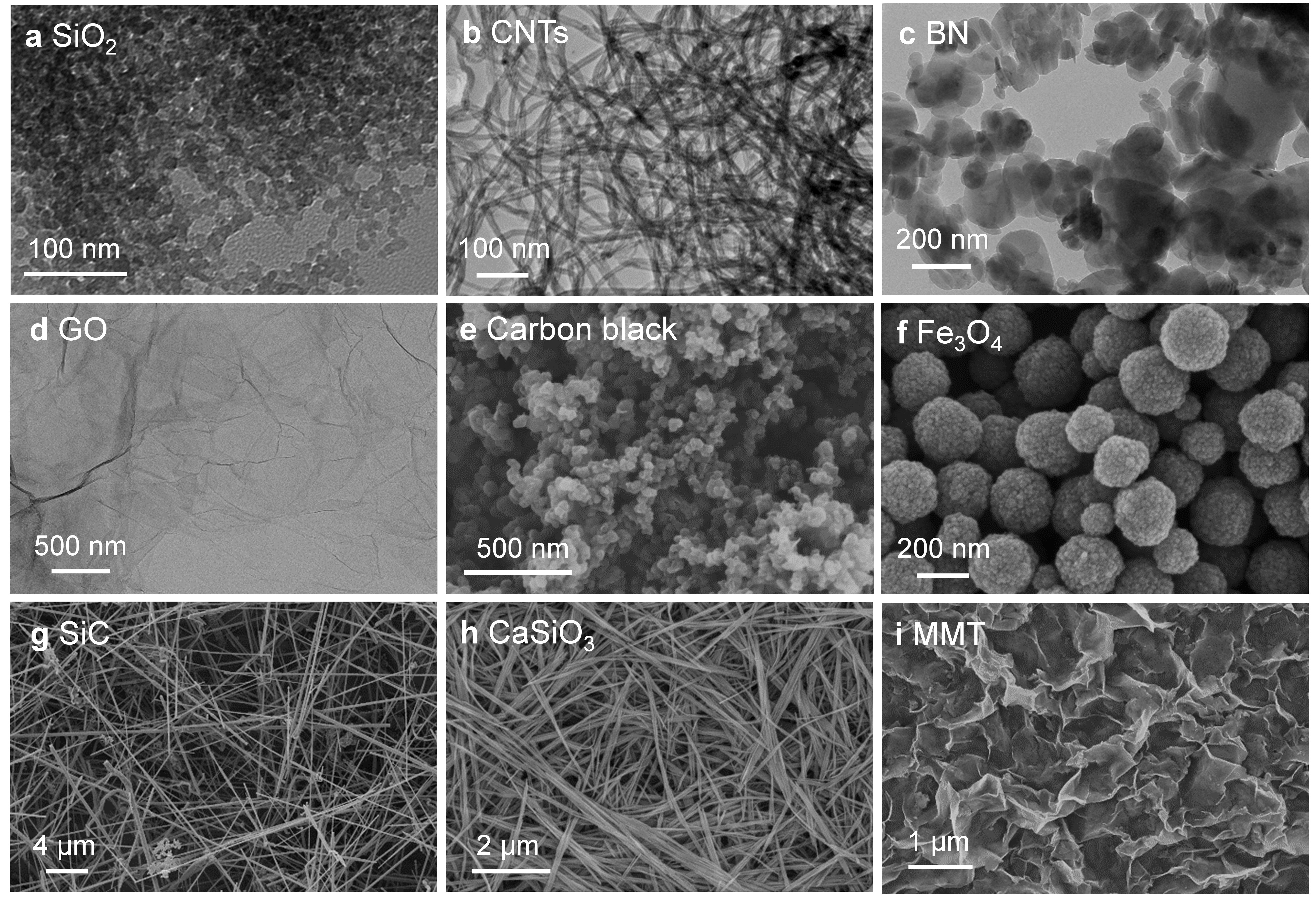


**Supplementary Figure 2. Morphologies of NBBs.** TEM images of silica nanoparticles (**a**), carbon nanotubes (**b**), boron nitride nanosheets (**c**), and GO (**d**). SEM image of carbon black nanoparticles (**e**), Fe_3_O_4_ nanoparticles (**f**), SiC nanowires (**g**), CaSiO_3_ nanowires (**h**) and MMT nanosheets **(i**).


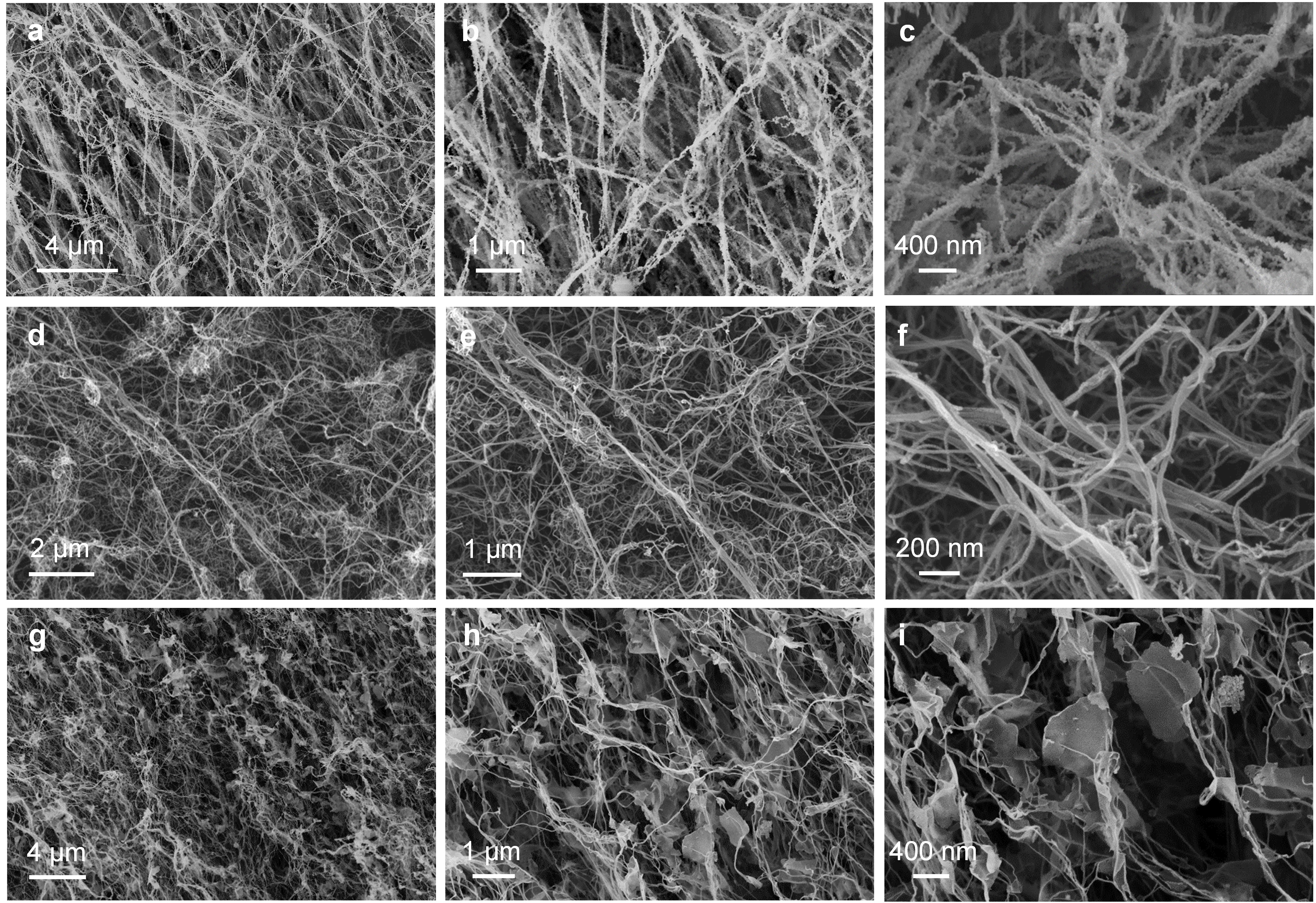


**Supplementary Figure 3. Additional SEM images of the biosynthesized nanocomposites. a** to **c,** SEM images of SiO_2_/BC at different magnifications. **d** to **f,** SEM images of CNTs/BC at different magnifications. **g** to **i,** SEM images of MMT/BC at different magnifications. These additional SEM images at different magnifications further confirmed the homogeneous distribution of NBBs over the whole samples.


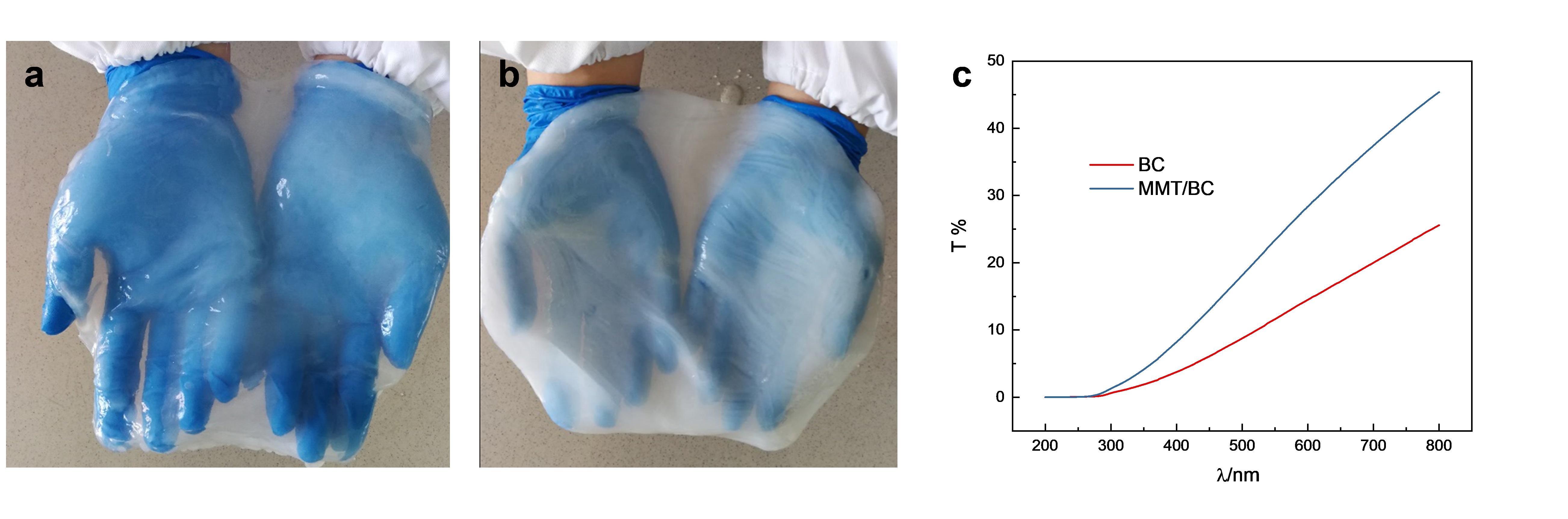


**Supplementary Figure 4. Comparison of the transparency between MMT/BC and pure BC.** **a,** Photograph of MMT/BC. **b,** Photograph of pure BC with the same thickness. **c,** Transmittance curve for MMT/BC film.


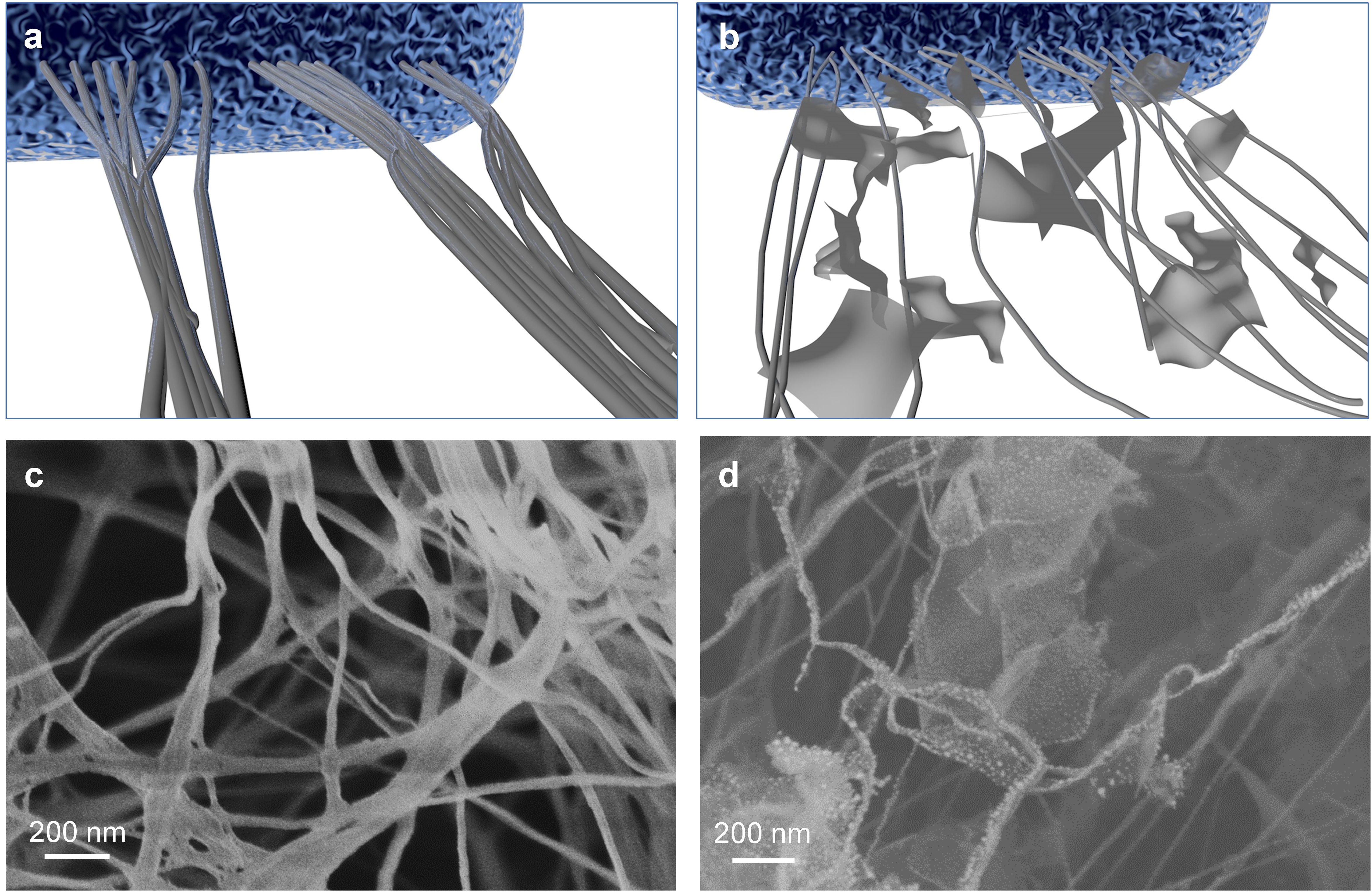


**Supplementary Figure 5. The addition of MMT interrupted the crystallization of BC and reduced the formation of wide ribbons.** Schematic of the formation process of pure BC (**a**) and MMT/BC (**b**). SEM image of pure BC (**c**) and MMT/BC (**d**).


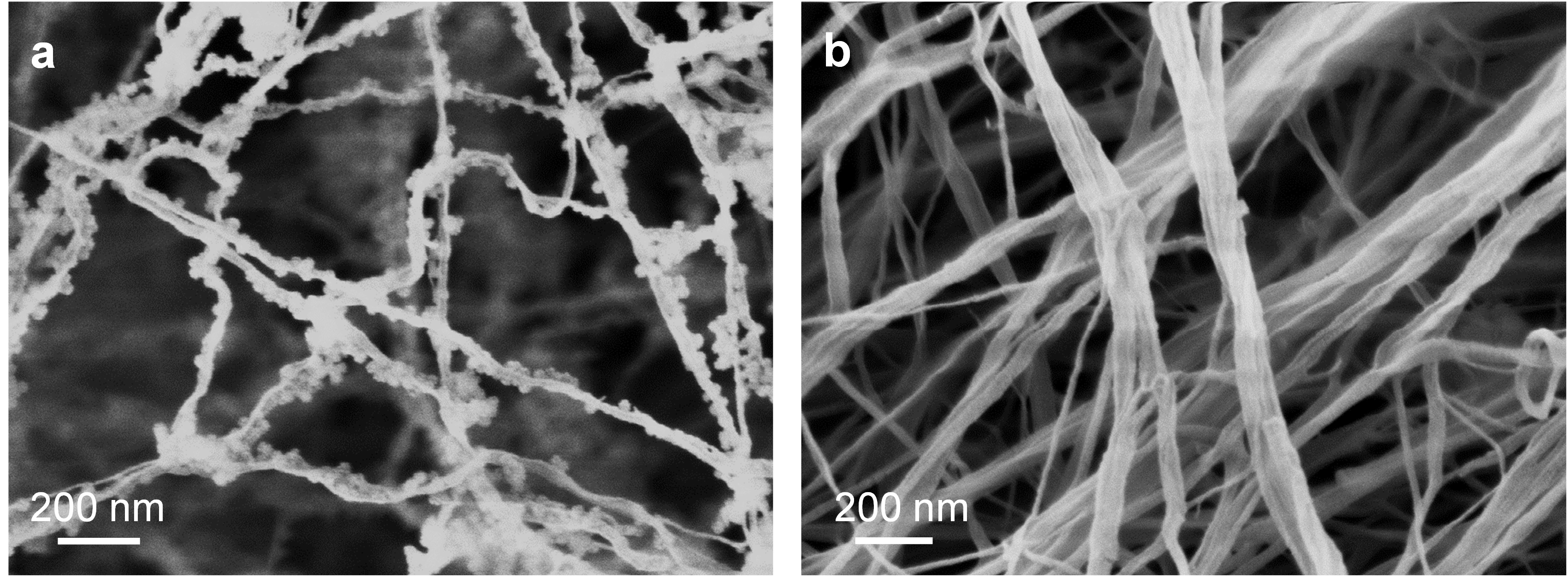


**Supplementary Figure 6. Comparison of the diameter of the cellulose nanofibrils in the biosynthesized SiO_2_/BC nanocomposite and pure BC. a,** SEM image of biosynthesized SiO_2_/BC. **b,** SEM image of pure BC. These SEM images clearly reveal much thinner cellulose nanofibrils in the SiO_2_/BC nanocomposites compared to pure BC, which further confirmed that SiO_2_ nanoparticles disrupted the bundling of microfibrils into ribbons during the biosynthesis.


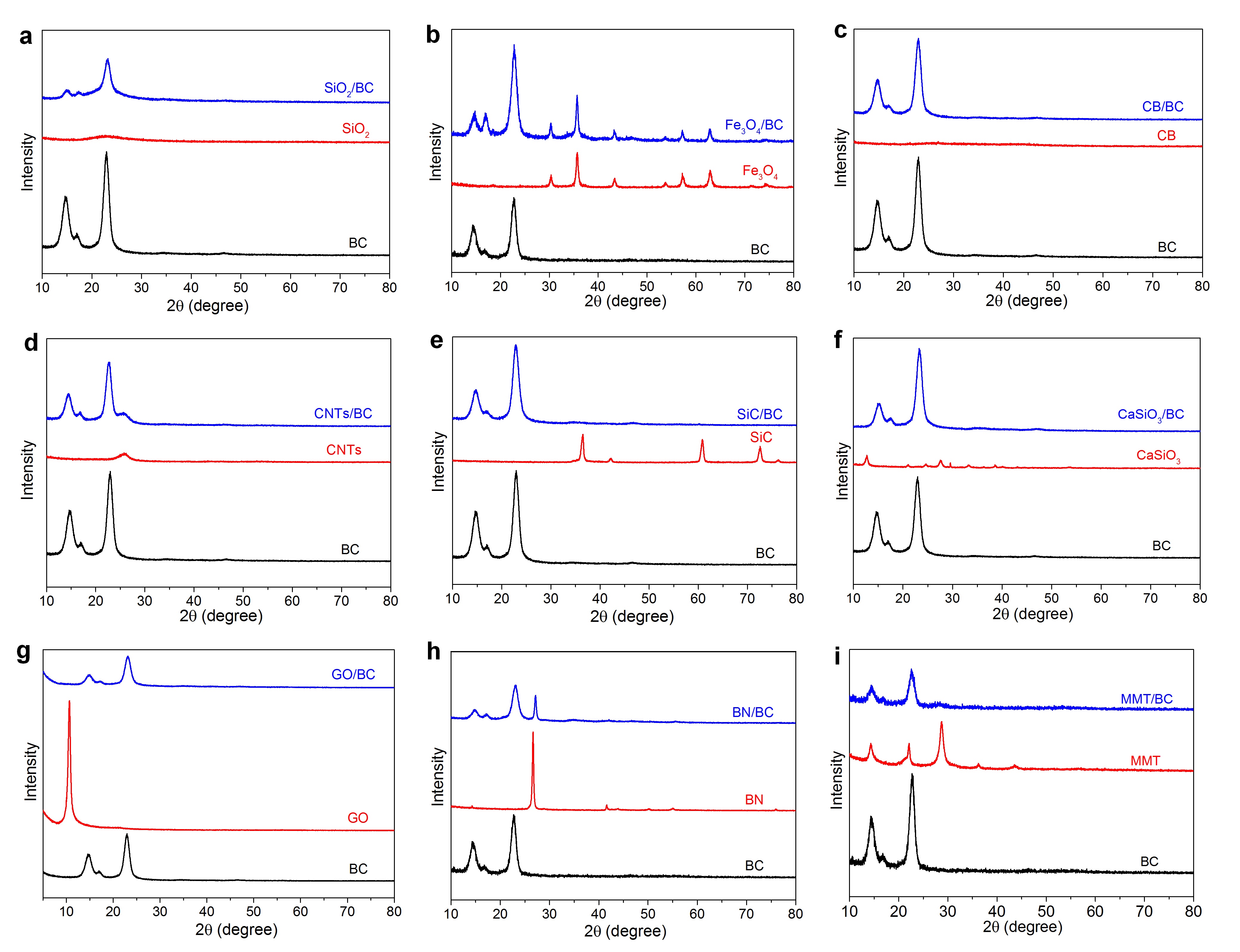


**Supplementary Figure 7. XRD patterns**. **a,** SiO_2_/BC. **b,** Fe_3_O_4_/BC. **c,** CB/BC. **d,** CNTs/BC. **e,** SiC/BC. **f,** CaSiO_3_/BC. **g,** GO/BC. **h,** BN/BC. **i,** MMT/BC.


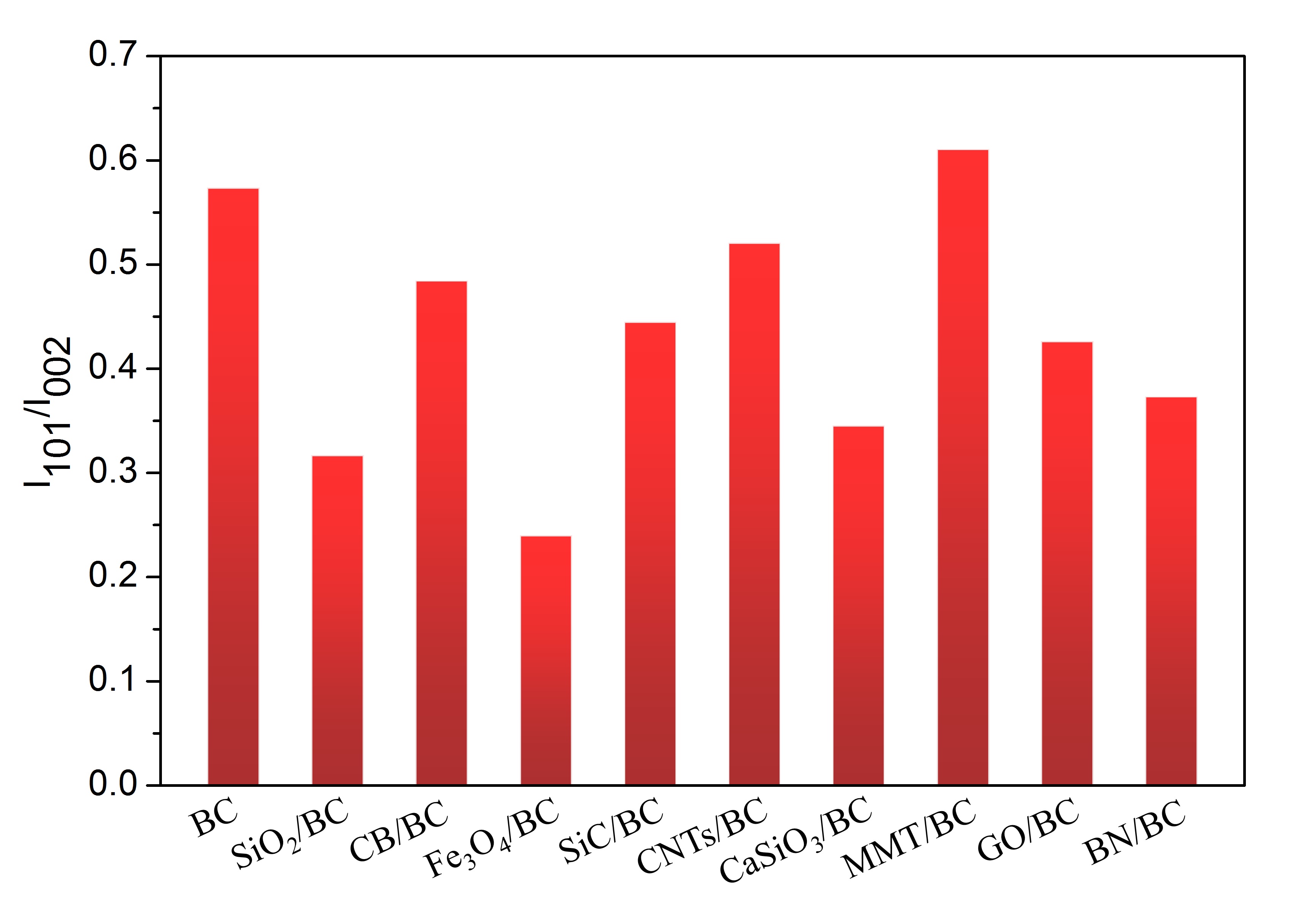


**Supplementary Figure 8. I_101_/I_002_ of the XRD patterns.** The height ratio of the (101) peak to the (002) peak for different nanocomposites. The (101) plane of the cellulose crystal is preferentially oriented parallel to the surface of the ribbon that are aligned parallel to the surface of the membrane. The height ratio of (101)/(002) peak of the biosynthesized nanocomposites is lower than the pure BC (except MMT/BC), indicating that NBBs disrupt the bundling of microfibrils into ribbons during the biosynthesis.


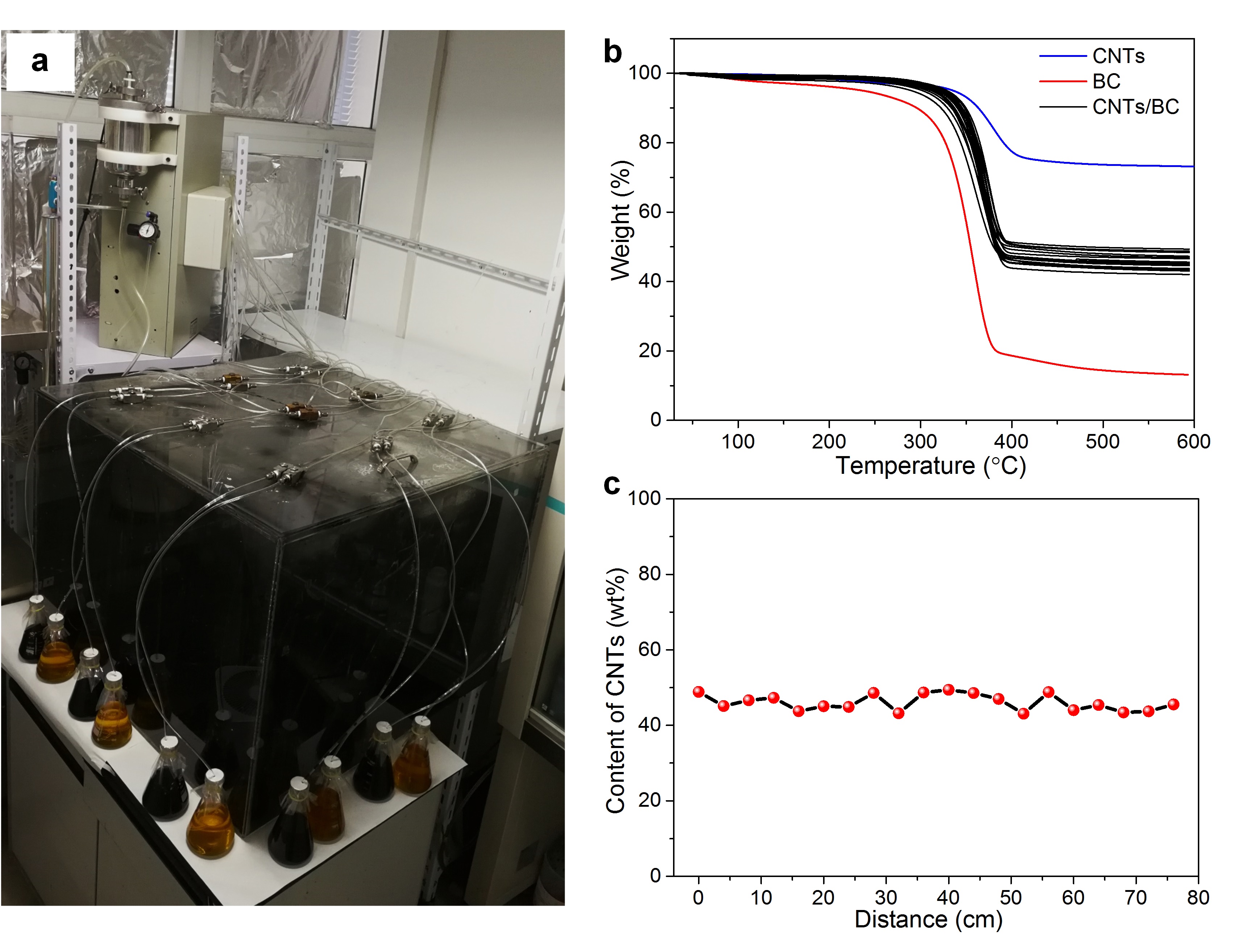


**Supplementary Figure 9. Large scale biosynthesis of CNTs/BC nanocomposites.** **a,** The large-scale biosynthesis apparatus. The bioreactor with the volume of 800×800×600 mm^3^ is equipped with 18 nozzles. **b,** TGA data of biosynthesized large-sized CNTs/BC nanocomposite (800 × 800 × 8 mm^3^). The samples were taken along the centerline every 4 cm for each TGA measurement. TGA curves of pure BC and CNTs are also shown in this figure. **c,** Contents of CNTs in the biosynthesized nanocomposite along the centerline, which was calculated based the TGA data.


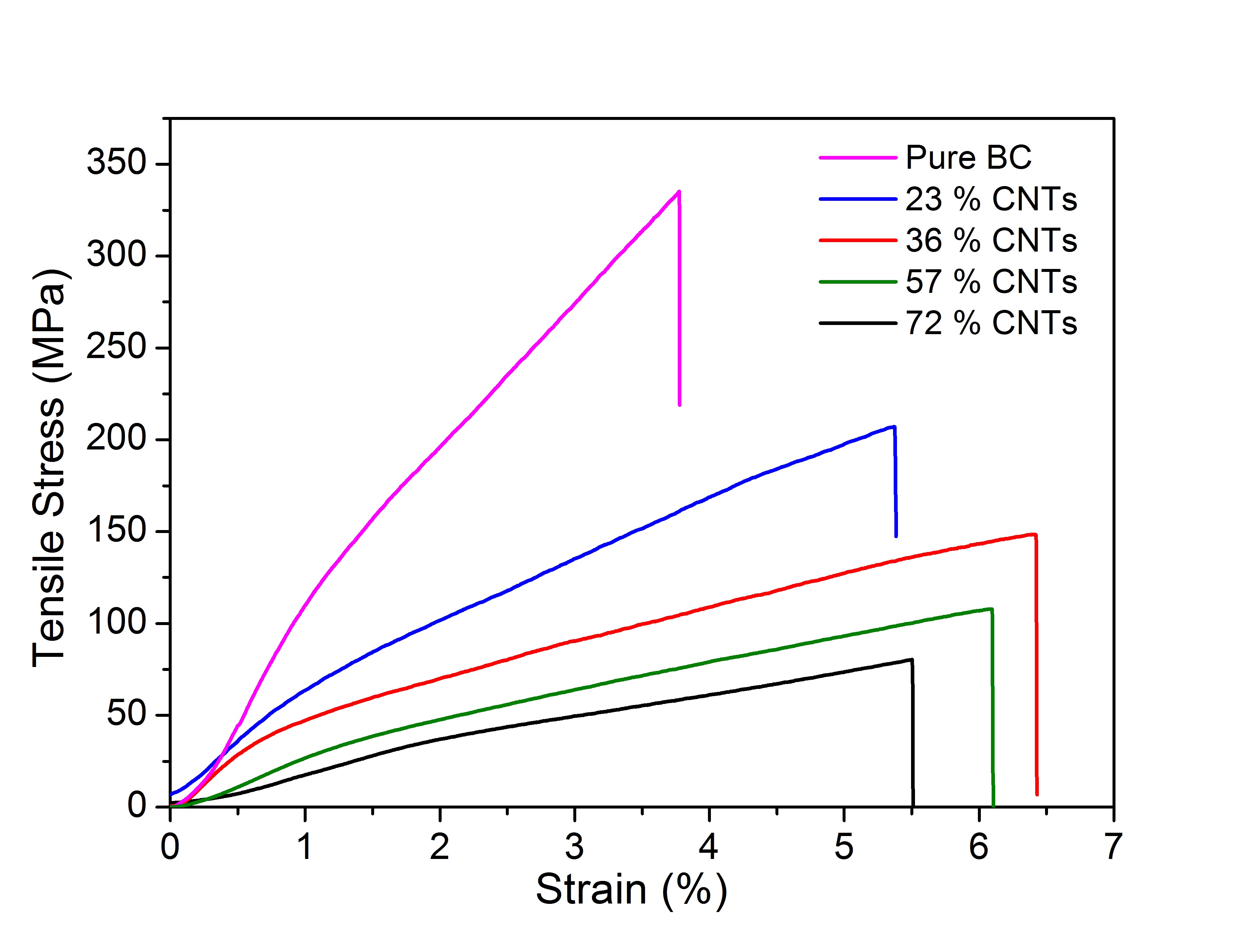


**Supplementary Figure 10. Mechanical properties of CNTs/BC nanocomposite films.** Tensile stress-strain curves of CNTs/BC nanocomposite films with various CNTs contents.


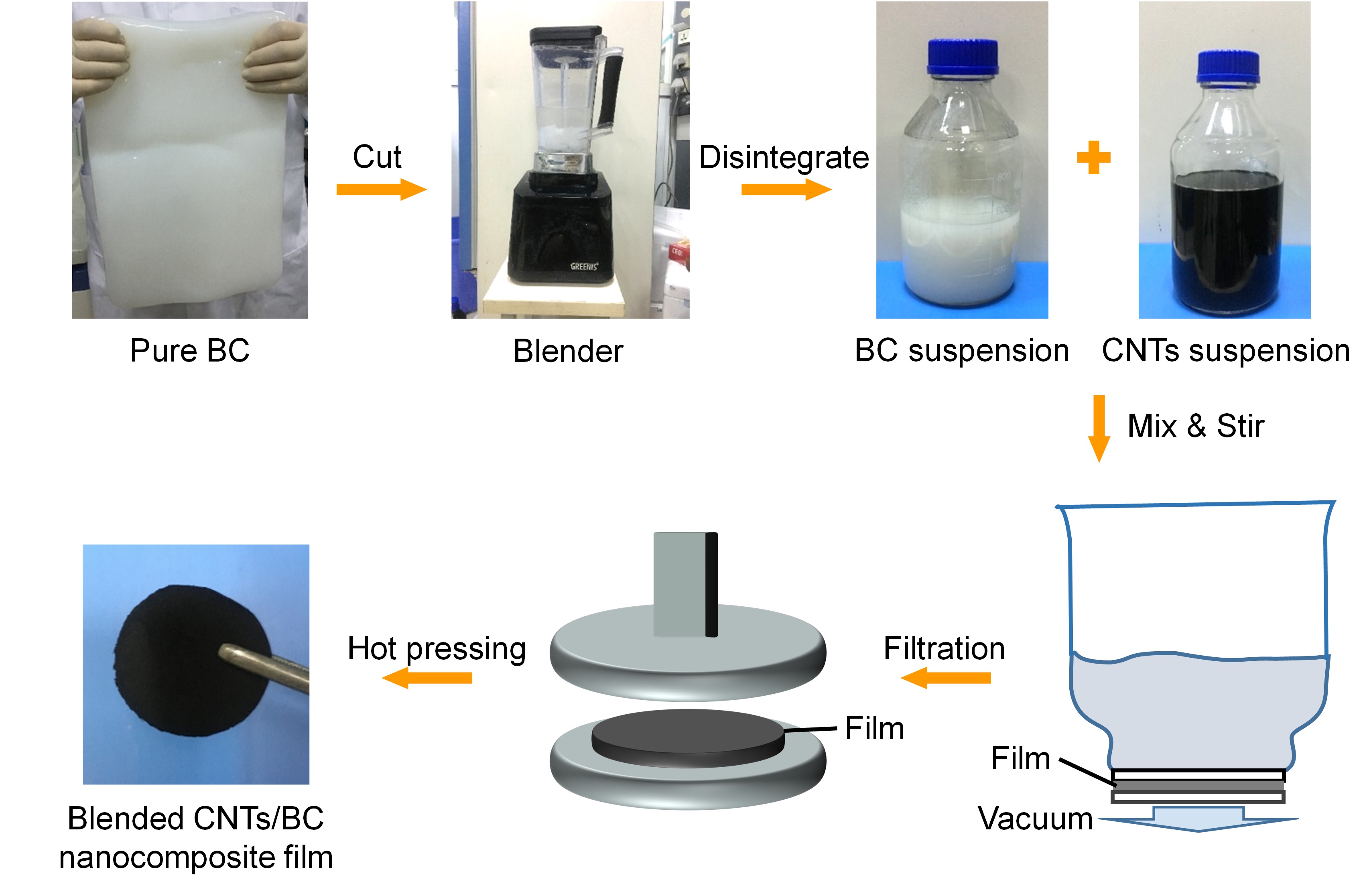


**Supplementary Figure 11. Schematic illustration of the process for preparing the blended CNTs/BC nanocomposites.** Pure BC pellicle was cut into pieces and disintegrated into a homogeneous suspension by a kitchen blender. Then the BC suspension was mixed with CNTs suspension under vigorous stirring to form a homogeneous mixture, which was finally vacuum filtered before hot-pressing under 100 MPa at 80 °C for 10 min.


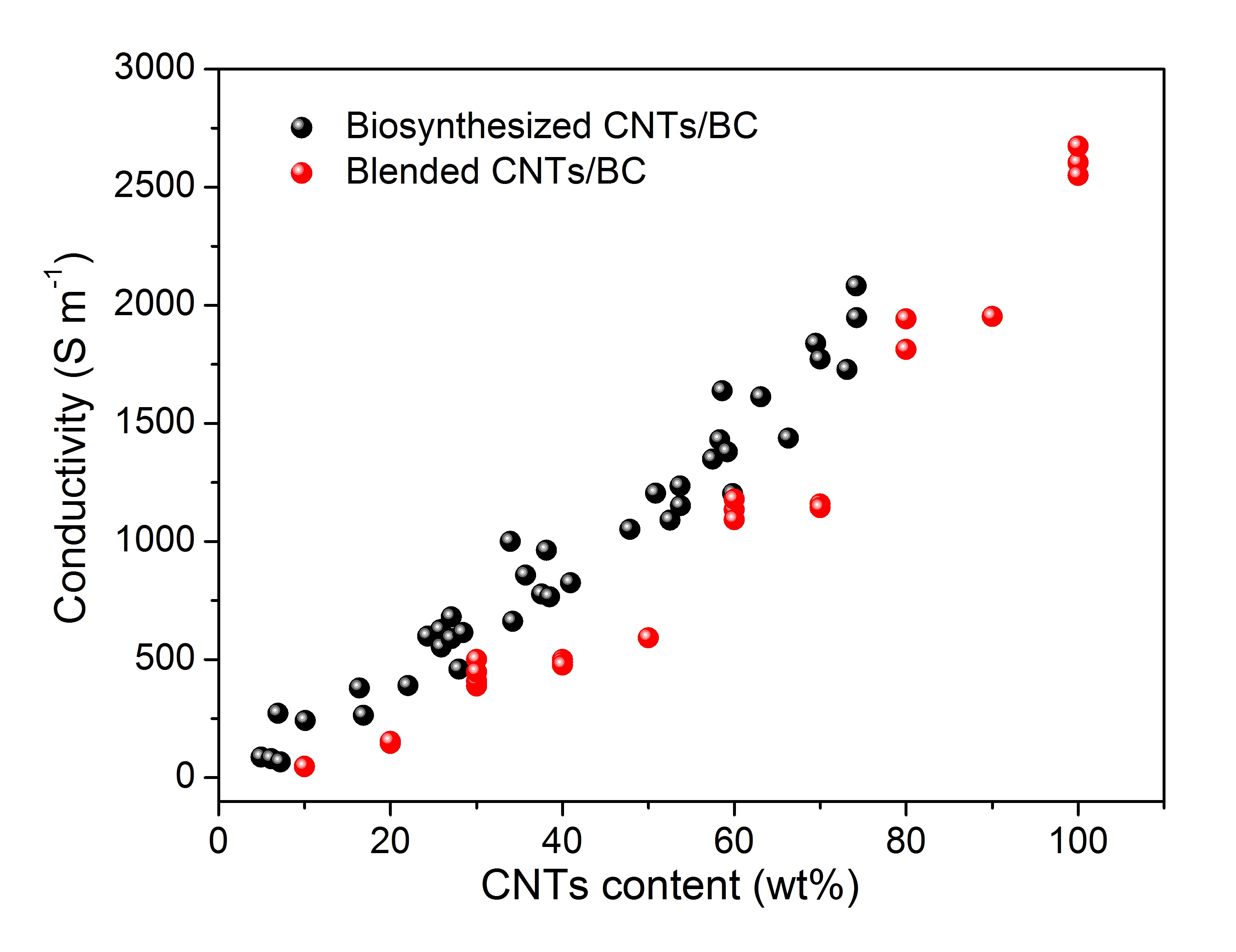


**Supplementary Figure 12. Comparison of the electrical conductivity between the biosynthesized and blended CNTs/BC nanocomposites.** The almost same electrical conductivity of the biosynthesized sample with the blended CNTs/BC nanocomposites indicates the highly uniform distribution of CNTs in the biosynthesized samples as well.


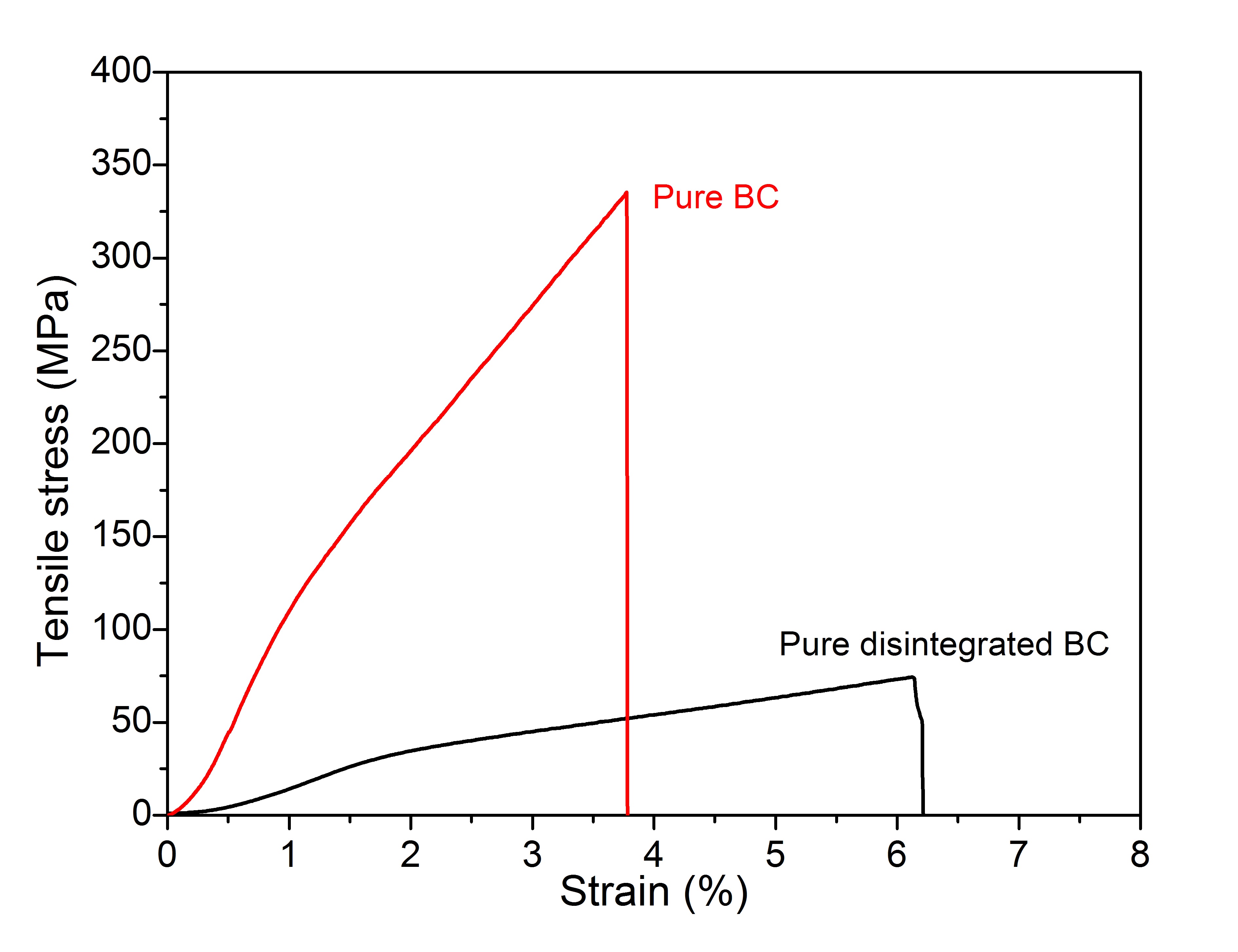


**Supplementary Figure 13. Comparison of the mechanical properties of pure BC and disintegrated BC films.** Tensile stress-strain curves of the directly hot-pressed pure BC film and the film sample fabricated from disintegrated BC suspension. The directly hot-pressed pure BC film exhibited a much higher tensile strength (335 MPa) than the disintegrated BC film (74 MPa). The extensive hydrogen bonding interaction as a result of the nano-size cellulose ribbons and the structural feature of ‘‘three-way branching points’’ in the as-biosynthesized BC contribute the high strength (1, 2). The disintegration treatment severely destroyed the 3D network structure and shortened the BC nanofibrils, which caused the decreased tensile strength for the disintegrated BC film(1). On the other hand, the disintegration treatment resulted in an increased ultimate tensile strain, presumably due to the fact that fragmented BC nanofibrils can more easily reorient themselves due to a weaker network structure (3).


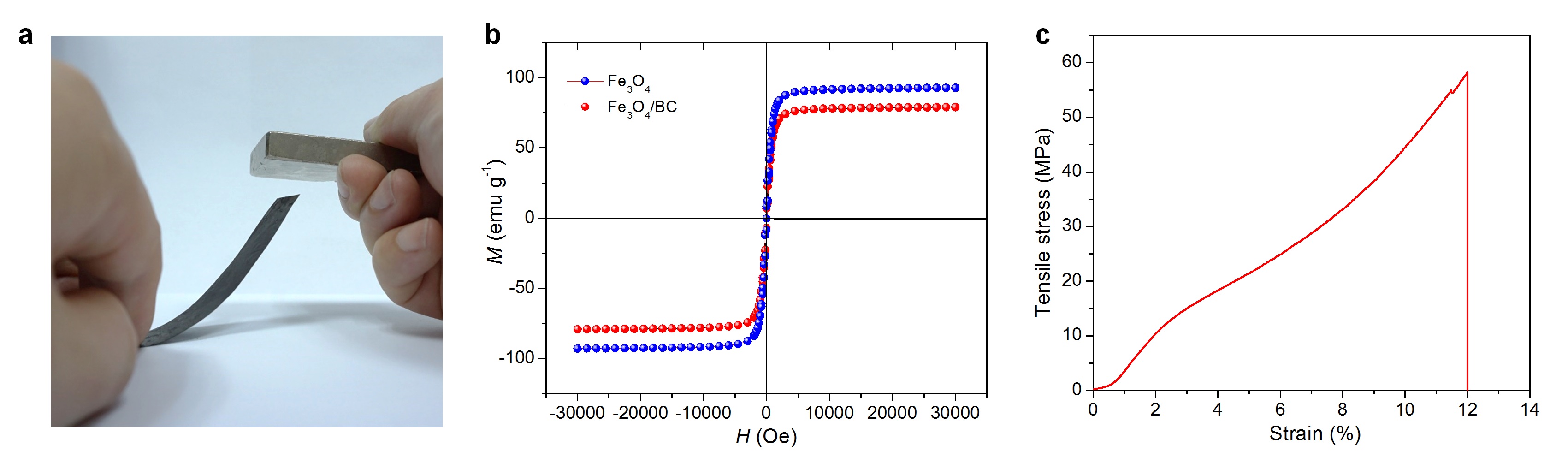


**Supplementary Figure 14. Magnetic Fe_3_O_4_/BC nanocomposite film.** **a,** The Fe_3_O_4_/BC nanocomposite film could be actuated by a simple household magnet. **b,** Room-temperature magnetic hysteresis loops of Fe_3_O_4_ nanoparticles and Fe_3_O_4_/BC nanocomposites, indicating a superparamagnetic behavior with a saturation magnetizations of 79 emu g^−1^. **c,** Tensile stress-strain curve of Fe_3_O_4_/BC nanocomposite film. The Fe_3_O_4_ content in the composite was 82 wt% according to the TGA analysis. The tensile strength of the biosynthesized Fe_3_O_4_/BC nanocomposites (58 MPa) was much high than that of cobalt ferrite nanoparticles/BC composites with similar nanoparticle weight fraction (82 wt%), which was fabricated with freeze-dried BC aerogels as templates(4). The flexible and high-strength magnetic Fe_3_O_4_/BC films are expected to be useful in various fields such as electromagnetic actuators, smart microfluidics devices, and biomedicine.

**Supplementary Tables**

**Supplementary Table 1.** Electrical conductivity and tensile strength of various polymer nanocomposites.

| **Type** | **Filler**  **[wt%]** | | **Matrix** | **σ**  **[S m^-1^]** | **Tensile strength**  **[MPa]** | **Ref** |
| --- | --- | --- | --- | --- | --- | --- |
| CNTs/polymer | 10 | | UHMWPE | 0.01 | 20 | (5) |
|  | 2 | Natural rubber | | 3.162E-7 | 29.6 | (6) |
|  | 10 | | PEO/ORC | 0.316 | 18 | (7) |
|  | 1.75 | | epoxy | 1 | 60 | (8) |
|  | 75 | | PLLA/PDLA | 1 | 27 | (9) |
|  | 20 | | PP | 0.2 | 27.5 | (10) |
|  | 5 | | Polystyrene | 12.5 | 30.6 | (11) |
|  | 3.5 | | PVDF | 0.01 | 60 | (12) |
|  | 1.5 | | LPC | 0.0316 | 70 | (13) |
|  | 70 | | PVA | 900 | 55 | (14) |
|  | 82 | | PVA | 1700 | 21 | (14) |
|  | 40 | | PVA | 20 | 44 | (14) |
|  | 60 | | PEO | 652 | 5 | (15) |
| rGO | 5 | | cellulose | 1E-3 | 200 | (16) |
|  | 80 | | PVA | 0.316 | 120 | (17) |
|  | 3.47 | | PS | 43.5 | 108 | (18) |
| Graphene/polymer | - | | PC | 417 | 130 | (19) |
| Blended CNTs/BC | 23 | | BC | 425.3 | 58.5 | This work |
|  | 36 | |  | 849.0 | 42.4 |  |
|  | 72 | |  | 1761.2 | 34.5 |  |
| **Biosynthesized CNTs/BC** | **23** | | **BC** | **495.53** | **200** | **This work** |
|  | **36** | |  | **832.2** | **145** |  |
|  | **72** | |  | **1765.1** | **80** |  |

Note: The single walled CNTs and aligned CNTs-based polymer nanocomposites were not included in this table.

**Supplementary Table 2.** EMI shielding performance of various carbon-based polymer nanocomposite shielding materials.

| **Type** | **Matrix** | **Filter**  **[wt%]** | **t**  **[mm]** | **σ**  **[S m**^-1^**]** | **SE**  **[dB]** | **F**  **[GHz]** | **Ref** |
| --- | --- | --- | --- | --- | --- | --- | --- |
| CNTs/polymer | Cellulose | 9.1 | 0.2 | 375 | 20 | 8-12 | (20) |
|  | Cellulose | 0.45^#^ | 0.01-0.02 | 1.8 | 20.8 | 8-12 | (21) |
|  | PU | 22 | 0.1 | 5 | 20 | 8-12 | (22) |
|  | PU | 10 | >0.2 | 12.4 | 29 | X-band | (23) |
|  | PU | 5 | 2 | 100 | 22 | X-band | (24) |
|  | PU/PEDOT | 30 | 2.5 | 275 | 45 | X-band | (25) |
|  | UHMWPE | 10 | 1 | 100 | 50 | 8-12.5 | (26) |
|  | PVDF | 3.5 | 1.1 | 100 | 17.7 | X-band | (27) |
|  | Epoxy | 15 | 1.5 | 15 | 30 | X-band | (28) |
|  | PMMA | 10^#^ | 2.1 | 150 | 40 | X-band | (29) |
|  | PMMA | 40 | 0.4 | 10 | 27 | 8-12 | (30) |
|  | ABS | 15 | 1.1 | 66.7 | 50 | 8-12 | (31) |
|  | Epoxy | 0.66 | 2 | 516 | 33 | 8-12 | (32) |
|  | PMMA | 40 | 0.165 | 1000 | 27 | 13.5 | (33) |
|  | WPU | 76.2 | 4.5 | 44.6 | 50 | 8-12 | (34) |
|  | PS | 20 | 2 |  | 30 | 8-12 | (35) |
|  | PC | 20 | 2.1 |  | 39 | 8-12 | (36) |
|  | PP | 7.5^#^ | 1 |  | 34.8 | X-band | (37) |
|  | PTT | 4.76^#^ | 2 | 4.5 | 22 | X-band | (38) |
|  | WPU | 76 | 0.05 | 2100 | 24 | 8-12 | (39) |
|  | WPU | 76 | 0.32 | 2100 | 49 | 8-12 | (39) |
|  | WPU | 76 | 0.8 | 2100 | 80 | 8-12 | (39) |
| rGO/polymer | PEI | 10 | 2.3 | 0.001 | 22 | 8-12 | (40) |
|  | PS | 30 | 2.0 | 1.25 | 29 | 8-12 | (41) |
|  | PI | 16 | 0.8 |  | 21 | 8-12 | (42) |
|  | Wax | 60^#^ | 0.35 | 2500 | 27 | 8-12 | (43) |
|  | EVA | 60 | 0.05 | 250 | 14 | 8-12 | (44) |
|  | EVA | 60 | 0.12 | 250 | 18 | 8-12 | (44) |
|  | EVA | 60 | 0.35 | 250 | 27 | 8-12 | (44) |
|  | PVA | 35 | 0.3 | <0.1 | 15 | 8-12 | (45) |
|  | Resin | 50 | 0.4 | 17000 | 41.8 | 8-12 | (46) |
|  | PVA | 10 | 1.8 | 0.0008 | 13 | 8-12 | (47) |
| Graphite/polymer | PE | 7.5^#^ | 2.5 | 10 | 51.6 | 8-12 | (48) |
|  | PE | 18.7^#^ | 3 |  | 33 | 8-12 | (49) |
|  | Epoxy | 2 | 5 | 2.6 | 11 | 8-12 | (50) |
|  | ABS | 15 | 3 | 16 | 60 | 8-12 | (51) |
| **Biosynthesized CNTs/BC** | **BC** | **54.1** | **0.042** | **1296** | **18.8** | **X-band** | **This work** |
|  |  |  | **0.098** |  | **36.5** |  |  |
|  |  |  | **0.292** |  | **46.6** |  |  |
|  |  |  | **0.430** |  | **60.4** |  |  |
|  |  |  | **0.610** |  | **75.2** |  |  |

^*^ X-band: 8.2-12.4 GHz.

^#^ Vol. %.

**Supplementary Table 3.** Specific EMI SE of various carbon-based polymer nanocomposite shielding materials.

| **Type** | **Matrix** | **Filter**  **[wt%]** | | **t**  **[mm]** | **SE**  **[dB]** | **SSE**  **[dB mc^2^ g^-1^]** | **SSE/t**  **[dB mc^2^ g^-1^]** | **Ref** |
| --- | --- | --- | --- | --- | --- | --- | --- | --- |
| CNTs/polymer | ABS | | 15 | 1.1 | 50 | 47.6 | 432.7 | (31) |
|  | PS | | 20 | 2 | 30 | 57 | 285 | (35) |
|  | PC | | 20 | 2.1 | 39 | 34.5 | 164 | (36) |
|  | WPU | | 76.2 | 1 | 21.1 | 541 | 5410 | (39) |
| rGO/polymer | PEI | | 10 | 2.3 | 12.8 | 44 | 191.3 | (40) |
|  | PS | | 30 | 2.0 | 29 | 64.4 | 257.6 | (41) |
|  | PEI | | 10 | 2.5 | 18 | 44 | 176 | (40) |
|  | PS | | 7 | 2.5 | 45.1 | 173 | 692 | (18) |
|  | PEDOT | | 25 | 0.8 | 70 | 67.3 | 841 | (52) |
| **Biosynthesized CNTs/BC** | **BC** | | **54.1** | **0.042** | **18.8** | **18.8** | **4476** | **This**  **work** |
|  |  |  |  | **0.098** | **36.5** | **36.5** | **3724** |  |
|  |  |  |  | **0.292** | **46.6** | **46.6** | **1596** |  |
|  |  |  |  | **0.430** | **60.4** | **60.4** | **1405** |  |
|  |  |  |  | **0.610** | **75.2** | **75.2** | **1233** |  |

Note: The density of the biosynthesized CNTs/BC film was about 1.0 g cm^-3^.

References

1. Yamanaka, S, Watanabe, K, Kitamura, N*, et al.* The structure and mechanical properties of sheets prepared from bacterial cellulose. *J Mater Sci*. 1989; **24**(9): 3141-5.

2. Soykeabkaew, N, Sian, C, Gea, S*, et al.* All-cellulose nanocomposites by surface selective dissolution of bacterial cellulose. *Cellulose*. 2009; **16**(3): 435-44.

3. Gea, S, Torres, FG, Troncoso, OP*, et al.* Biocomposites based on bacterial cellulose and apple and radish pulp. *Int Polym Proc*. 2007; **22**(5): 497-501.

4. Olsson, RT, Samir, M, Salazar-Alvarez, G*, et al.* Making flexible magnetic aerogels and stiff magnetic nanopaper using cellulose nanofibrils as templates. *Nat Nanotechnol*. 2010; **5**(8): 584-8.

5. Deplancke, T, Lame, O, Barrau, S*, et al.* Impact of carbon nanotube prelocalization on the ultra-low electrical percolation threshold and on the mechanical behavior of sintered UHMWPE-based nanocomposites. *Polymer*. 2017; **111**: 204-13.

6. George, N, Bipinbal, PK, Bhadran, B*, et al.* Segregated network formation of multiwalled carbon nanotubes in natural rubber through surfactant assisted latex compounding: A novel technique for multifunctional properties. *Polymer*. 2017; **112**: 264-77.

7. Gong, T, Liu, M-Q, Liu, H*, et al.* Selective distribution and migration of carbon nanotubes enhanced electrical and mechanical performances in polyolefin elastomers. *Polymer*. 2017; **110**: 1-11.

8. Hawkins, SA, Yao, H, Wang, H*, et al.* Tensile properties and electrical conductivity of epoxy composite thin films containing zinc oxide quantum dots and multi-walled carbon nanotubes. *Carbon*. 2017; **115**: 18-27.

9. Liu, Z, Bai, H, Luo, Y*, et al.* Achieving a low electrical percolation threshold and superior mechanical performance in poly(L-lactide)/thermoplastic polyurethane/carbon nanotubes composites via tailoring phase morphology with the aid of stereocomplex crystallites. *RSC Adv*. 2017; **7**(18): 11076-84.

10. Andrews, R, Jacques, D, Minot, M*, et al.* Fabrication of carbon multiwall nanotube/polymer composites by shear mixing. *Macromol Mater Eng*. 2002; **287**(6): 395-403.

11. Safadi, B, Andrews, R, Grulke, E. Multiwalled carbon nanotube polymer composites: synthesis and characterization of thin films. *J Appl Polym Sci*. 2002; **84**(14): 2660-9.

12. Kumar, GS, Vishnupriya, D, Chary, KS*, et al.* High dielectric permittivity and improved mechanical and thermal properties of poly (vinylidene fluoride) composites with low carbon nanotube content: effect of composite processing on phase behavior and dielectric properties. *Nanotechnology*. 2016; **27**(38): 385702.

13. Gao, X, Isayev, AI, Yi, C. Ultrasonic treatment of polycarbonate/carbon nanotubes composites. *Polymer*. 2016; **84**: 209-22.

14. Bartholome, C, Derre, A, Roubeau, O*, et al.* Electromechanical properties of nanotube-PVA composite actuator bimorphs. *Nanotechnology*. 2008; **19**(32): 325501.

15. Awasthi, K, Awasthi, S, Srivastava, A*, et al.* Synthesis and characterization of carbon nanotube-polyethylene oxide composites. *Nanotechnology*. 2006; **17**(21): 5417-22.

16. Feng, Y, Zhang, X, Shen, Y*, et al.* A mechanically strong, flexible and conductive film based on bacterial cellulose/graphene nanocomposite. *Carbohydr Polym*. 2012; **87**(1): 644-9.

17. Li, YQ, Yu, T, Yang, TY*, et al.* Bio‐inspired nacre‐like composite films based on graphene with superior mechanical, electrical, and biocompatible properties. *Adv Mater*. 2012; **24**(25): 3426-31.

18. Yan, DX, Pang, H, Li, B*, et al.* Structured Reduced Graphene Oxide/Polymer Composites for Ultra‐Efficient Electromagnetic Interference Shielding. *Adv Funct Mater*. 2015; **25**(4): 559-66.

19. Liu, P, Jin, Z, Katsukis, G*, et al.* Layered and scrolled nanocomposites with aligned semi-infinite graphene inclusions at the platelet limit. *Science*. 2016; **353**(6297): 364-7.

20. Imai, M, Akiyama, K, Tanaka, T*, et al.* Highly strong and conductive carbon nanotube/cellulose composite paper. *Compos Sci Technol*. 2010; **70**(10): 1564-70.

21. Yang, Y, Gupta, MC, Dudley, KL*, et al.* Conductive carbon nanofiber–polymer foam structures. *Adv Mater*. 2005; **17**(16): 1999-2003.

22. Li, Y, Chen, C, Zhang, S*, et al.* Electrical conductivity and electromagnetic interference shielding characteristics of multiwalled carbon nanotube filled polyacrylate composite films. *Appl Surf Sci*. 2008; **254**(18): 5766-71.

23. Gupta, TK, Singh, BP, Dhakate, SR*, et al.* Improved nanoindentation and microwave shielding properties of modified MWCNT reinforced polyurethane composites. *J Mater Chem A*. 2013; **1**(32): 9138-49.

24. Liu, Z, Bai, G, Huang, Y*, et al.* Microwave absorption of single-walled carbon nanotubes/soluble cross-linked polyurethane composites. *J Phys Chem C*. 2007; **111**(37): 13696-700.

25. Farukh, M, Dhawan, R, Singh, BP*, et al.* Sandwich composites of polyurethane reinforced with poly (3, 4-ethylene dioxythiophene)-coated multiwalled carbon nanotubes with exceptional electromagnetic interference shielding properties. *RSC Adv*. 2015; **5**(92): 75229-38.

26. Farukh, M, Singh, AP, Dhawan, S. Enhanced electromagnetic shielding behavior of multi-walled carbon nanotube entrenched poly (3, 4-ethylenedioxythiophene) nanocomposites. *Compos Sci Technol*. 2015; **114**: 94-102.

27. Al-Saleh, MH. Influence of conductive network structure on the EMI shielding and electrical percolation of carbon nanotube/polymer nanocomposites. *Synth Met*. 2015; **205**: 78-84.

28. Singh, AP, Gupta, BK, Mishra, M*, et al.* Multiwalled carbon nanotube/cement composites with exceptional electromagnetic interference shielding properties. *Carbon*. 2013; **56**: 86-96.

29. Li, N, Huang, Y, Du, F*, et al.* Electromagnetic interference (EMI) shielding of single-walled carbon nanotube epoxy composites. *Nano Lett*. 2006; **6**(6): 1141-5.

30. Kim, H, Kim, K, Lee, S*, et al.* Charge transport properties of composites of multiwalled carbon nanotube with metal catalyst and polymer: application to electromagnetic interference shielding. *Curr Appl Phys*. 2004; **4**(6): 577-80.

31. Al-Saleh, MH, Saadeh, WH, Sundararaj, U. EMI shielding effectiveness of carbon based nanostructured polymeric materials: a comparative study. *Carbon*. 2013; **60**: 146-56.

32. Chen, Y, Zhang, HB, Yang, Y*, et al.* High‐Performance Epoxy Nanocomposites Reinforced with Three‐Dimensional Carbon Nanotube Sponge for Electromagnetic Interference Shielding. *Adv Funct Mater*. 2016; **26**(3): 447-55.

33. Kim, HM, Kim, K, Lee, CY*, et al.* Electrical conductivity and electromagnetic interference shielding of multiwalled carbon nanotube composites containing Fe catalyst. *Appl Phys Lett*. 2004; **84**(4): 589-91.

34. Zeng, Z, Jin, H, Chen, M*, et al.* Lightweight and anisotropic porous MWCNT/WPU composites for ultrahigh performance electromagnetic interference shielding. *Adv Funct Mater*. 2016; **26**(2): 303-10.

35. Arjmand, M, Apperley, T, Okoniewski, M*, et al.* Comparative study of electromagnetic interference shielding properties of injection molded versus compression molded multi-walled carbon nanotube/polystyrene composites. *Carbon*. 2012; **50**(14): 5126-34.

36. Pande, S, Chaudhary, A, Patel, D*, et al.* Mechanical and electrical properties of multiwall carbon nanotube/polycarbonate composites for electrostatic discharge and electromagnetic interference shielding applications. *RSC Adv*. 2014; **4**(27): 13839-49.

37. Al-Saleh, MH, Sundararaj, U. Electromagnetic interference shielding mechanisms of CNT/polymer composites. *Carbon*. 2009; **47**(7): 1738-46.

38. Gupta, A, Choudhary, V. Electrical conductivity and shielding effectiveness of poly(trimethylene terephthalate)/multiwalled carbon nanotube composites. *J Mater Sci*. 2011; **46**(19): 6416-23.

39. Zeng, Z, Chen, M, Jin, H*, et al.* Thin and flexible multi-walled carbon nanotube/waterborne polyurethane composites with high-performance electromagnetic interference shielding. *Carbon*. 2016; **96**: 768-77.

40. Ling, J, Zhai, W, Feng, W*, et al.* Facile preparation of lightweight microcellular polyetherimide/graphene composite foams for electromagnetic interference shielding. *ACS Appl Mater Interfaces*. 2013; **5**(7): 2677-84.

41. Yan, DX, Ren, PG, Pang, H*, et al.* Efficient electromagnetic interference shielding of lightweight graphene/polystyrene composite. *J Mater Chem*. 2012; **22**(36): 18772-4.

42. Li, Y, Pei, X, Shen, B*, et al.* Polyimide/graphene composite foam sheets with ultrahigh thermostability for electromagnetic interference shielding. *RSC Adv*. 2015; **5**(31): 24342-51.

43. Yuan, B, Bao, C, Qian, X*, et al.* Design of artificial nacre-like hybrid films as shielding to mitigate electromagnetic pollution. *Carbon*. 2014; **75**: 178-89.

44. Song, W-L, Cao, M-S, Lu, M-M*, et al.* Flexible graphene/polymer composite films in sandwich structures for effective electromagnetic interference shielding. *Carbon*. 2014; **66**: 67-76.

45. Rao, BB, Yadav, P, Aepuru, R*, et al.* Single-layer graphene-assembled 3D porous carbon composites with PVA and Fe 3 O 4 nano-fillers: an interface-mediated superior dielectric and EMI shielding performance. *Phys Chem Chem Phys*. 2015; **17**(28): 18353-63.

46. Singh, AP, Garg, P, Alam, F*, et al.* Phenolic resin-based composite sheets filled with mixtures of reduced graphene oxide, γ-Fe 2 O 3 and carbon fibers for excellent electromagnetic interference shielding in the X-band. *Carbon*. 2012; **50**(10): 3868-75.

47. Yao, K, Gong, J, Tian, N*, et al.* Flammability properties and electromagnetic interference shielding of PVC/graphene composites containing Fe 3 O 4 nanoparticles. *RSC Adv*. 2015; **5**(40): 31910-9.

48. Jiang, X, Yan, D-X, Bao, Y*, et al.* Facile, green and affordable strategy for structuring natural graphite/polymer composite with efficient electromagnetic interference shielding. *RSC Adv*. 2015; **5**(29): 22587-92.

49. Panwar, V, Mehra, R. Analysis of electrical, dielectric, and electromagnetic interference shielding behavior of graphite filled high density polyethylene composites. *Polym Eng Sci*. 2008; **48**(11): 2178-87.

50. De Bellis, G, Tamburrano, A, Dinescu, A*, et al.* Electromagnetic properties of composites containing graphite nanoplatelets at radio frequency. *Carbon*. 2011; **49**(13): 4291-300.

51. Sachdev, V, Patel, K, Bhattacharya, S*, et al.* Electromagnetic interference shielding of graphite/acrylonitrile butadiene styrene composites. *J Appl Polym Sci*. 2011; **120**(2): 1100-5.

52. Agnihotri, N, Chakrabarti, K, De, A. Highly efficient electromagnetic interference shielding using graphite nanoplatelet/poly (3, 4-ethylenedioxythiophene)–poly (styrenesulfonate) composites with enhanced thermal conductivity. *RSC Adv*. 2015; **5**(54): 43765-71.
